# Mycorrhizal phosphate uptake pathway in maize: vital for growth and cob development on nutrient poor agricultural and greenhouse soils

**DOI:** 10.3389/fpls.2013.00533

**Published:** 2013-12-26

**Authors:** Martin Willmann, Nina Gerlach, Benjamin Buer, Aleksandra Polatajko, Réka Nagy, Eva Koebke, Jan Jansa, René Flisch, Marcel Bucher

**Affiliations:** ^1^Botanical Institute, University of CologneCologne, Germany; ^2^Cluster of Excellence on Plant Sciences (CEPLAS), University of CologneCologne, Germany; ^3^Experimental Station Eschikon, Institute of Agricultural Sciences, Federal Institute of Technology Zurich (ETH)Lindau, Switzerland; ^4^Agroscope Reckenholz-Tänikon Research Station ARTZurich, Switzerland

**Keywords:** *Zea mays*, phosphate transporter, phosphorus nutrition, arbuscular mycorrhiza, plant biomass

## Abstract

Arbuscular mycorrhizal fungi (AMF) form a mutually beneficial symbiosis with plant roots providing predominantly phosphorus in the form of orthophosphate (Pi) in exchange for plant carbohydrates on low P soils. The goal of this work was to generate molecular-genetic evidence in support of a major impact of the mycorrhizal Pi uptake (MPU) pathway on the productivity of the major crop plant maize under field and controlled conditions. Here we show, that a loss-of-function mutation in the mycorrhiza-specific Pi transporter gene *Pht1;6* correlates with a dramatic reduction of above-ground biomass and cob production in agro-ecosystems with low P soils. In parallel mutant *pht1;6* plants exhibited an altered fingerprint of chemical elements in shoots dependent on soil P availability. In controlled environments mycorrhiza development was impaired in mutant plants when grown alone. The presence of neighboring mycorrhizal nurse plants enhanced the reduced mycorrhiza formation in *pht1;6* roots. Uptake of ^33^P-labeled orthophosphate via the MPU pathway was strongly impaired in colonized mutant plants. Moreover, repression of the MPU pathway resulted in a redirection of Pi to neighboring plants. In line with previous results, our data highlight the relevance of the MPU pathway in Pi allocation within plant communities and in particular the role of Pht1;6 for the establishment of symbiotic Pi uptake and for maize productivity and nutritional value in low-input agricultural systems. In a first attempt to identify cellular pathways which are affected by Pht1;6 activity, gene expression profiling via RNA-Seq was performed and revealed a set of maize genes involved in cellular signaling which exhibited differential regulation in mycorrhizal *pht1;6* and control plants. The RNA data provided support for the hypothesis that fungal supply of Pi and/or Pi transport across Pht1;6 affects cell wall biosynthesis and hormone metabolism in colonized root cells.

## Introduction

Phosphorus (P) is a nutrient which frequently is not readily available to plants thus limiting biomass production in marine, freshwater, and terrestrial ecosystems (Elser et al., 2007; Carpenter and Bennett, 2011). Even when soil P levels exceed plant requirements more than 100-fold (Bieleski, 1973; Schachtman et al., 1998) P may still limit plant growth because the element is mainly present in immobile forms that are not directly available to the majority of crop plants (Marschner, 1995). Large amounts of P fertilizer are applied each year to ensure high yields in intensive agricultural, horticultural or biofuel productions (Cordell et al., 2009; Macdonald et al., 2011), which frequently causes eutrophication of water bodies (Bennett et al., 2001; Smith and Schindler, 2009). The demand for P is expected to exceed its global availability within only a few decades (Cordell et al., 2009; Vaccari and Strigul, 2011). To allow growth under low P conditions plants developed, >400 Million years ago (Redecker et al., 2000), a powerful strategy that resulted in an extended nutrient absorptive surface area through formation of arbuscular mycorrhiza (AM; see for example Parniske, 2008; Smith and Read, 2008). AM is a symbiotic association of plant roots with fungi of the phylum *Glomeromycota* (Schüßler et al., 2001). At the cost of photosynthetic carbon, the benefit for the mycorrhizal host plant is mainly a more efficient uptake of orthophosphate (Pi) delivered by the extensive fungal network (Pearson and Jakobsen, 1993a; Bucher, 2007; Smith and Read, 2008). Extraradical hyphae of AM can deliver most of total plant P via the “mycorrhizal Pi uptake (MPU) pathway” (Pearson and Jakobsen, 1993b; Smith et al., 2003, 2004). In MPU Pi is unloaded from myco- to photobiont in colonized root cortex cells in which fungal hyphae form hyphal coils and arbuscules formed by repeated dichotomous branching of the fungal hypha. Here Pi leaves the hypha into the peri-arbuscular space by an as yet unknown mechanism where it is absorbed by the colonized cortex cell.

More than 30 years ago, biochemical data on Pi uptake in plants had provided evidence for H^+^/Pi co-transport systems dependent on the activity of a proton extrusion pump (Ullrich-Eberius et al., 1981). In attempts to elucidate the molecular underpinnings of Pi transport, the first plant Pi transporter genes had been identified in *Arabidopsis* and potato encoding proteins of the Pht1 family of H^+^/Pi co-transporters (Muchhal et al., 1996; Leggewie et al., 1997). Subsequently mycorrhiza-specific Pht1 transporters were shown to be expressed in mycorrhizal roots of potato, *Medicago truncatula* and monocot rice (Rausch et al., 2001; Harrison et al., 2002; Paszkowski et al., 2002) and later in maize and other cereals (Glassop et al., 2005; Nagy et al., 2006). Immunolocalization and expression studies on the *Medicago* protein MtPT4 demonstrated subcellular targeting of the transporter to the plant peri-arbuscular membrane (Harrison et al., 2002; Pumplin et al., 2012), the site of symbiotic nutrient exchange between both symbiotic partners.

The mutualistic symbiosis of plant roots with AM fungi is a complex trait resulting in cooperative interactions among the two symbionts including bi-directional exchange of signaling molecules and metabolic resources. The molecular mechanisms underlying the establishment of symbiosis are under intense study, yet little is known about regulation of the MPU pathway. It is well established that maize is highly responsive to AM colonization especially when it is grown at low available Pi (Khan, 1972; Kaeppler et al., 2000). Reverse plant genetics has allowed detailed studies under controlled conditions on Pi transporter mutants which are impaired in mycorrhizal Pi transport at the peri-arbuscular membrane. These Pi transporter mutants are confined to two model species of the Fabaceae family, i.e., *Lotus japonicus* and *Medicago truncatula* (Maeda et al., 2006; Javot et al., 2007), and rice (Yang et al., 2012). However, mechanistic evidence in support of a major impact of the activity of a specific Pht1 protein on AM symbiosis development, MPU pathway activity and its contribution to the productivity of crop plants under field conditions is currently lacking. Pht1 gene expression studies gave support to the role of encoded proteins in P nutrition in the agricultural setting (Nagy et al., 2006; Yang et al., 2012). AM symbiosis improved growth of rice plants in controlled and field conditions (Solaiman and Hirata, 1997), but the role of the MPU pathway in dry and wetland rice fields is unclear due to absence of clear genetic evidence (Anderson et al., 1986; Yang et al., 2012) and targeted reverse genetics approaches are needed. Although the two legume species are ideal model plants for functional genomics studies, they are of limited economical value, and consequences of the root nodule symbiosis with nitrogen-fixing bacteria might obscure effects of AM symbiosis, especially in field conditions. It has repeatedly been described in the literature, that co-inoculation with rhizobia and AM fungi significantly affected host plant physiology under low P and/or low N conditions for example as a result of intersymbiont competition for P and photosynthate in tripartite legume/*Rhizobium*/AM fungal associations (Paul and Kucey, 1981; Bethlenfalvay et al., 1982; Ballesteros-Almanza et al., 2010; Tavasolee et al., 2011; Wang et al., 2011). The aim of this work was to investigate the role of a plant gene encoding a protein with a central role in AM symbiosis function in the field and under controlled experimental conditions in the greenhouse and to study commonalities and potential phenotypical differences between the two settings. We worked with a MPU pathway-loss-of-function transposon insertion mutant in maize, one of the world's major crops, under laboratory and field conditions, without the complications arising from the use of transgenic plants. The maize Pi transporter gene *ZEAma;Pht1;6* (also named ZmPT6 or *Pht1;6*) was suggested to be involved in the MPU pathway in mycorrhizal maize roots, based on expression analyses and transcript localization to arbuscule-containing cells (Glassop et al., 2005; Nagy et al., 2006). In addition, phylogenetic clustering revealed high sequence homology of Pht1;6 protein with other mycorrhiza-specific Pi transporters from dicot and monocot species. The study of a *Pht1;6* mutant which exhibits impaired MPU pathway activity provided insight into the phenotypic impact of the MPU pathway in maize when cultivated under agricultural field conditions and how neighboring plants might have contributed to successful AM fungal colonization of mutant roots. Moreover, *Pht1;6* loss-of-function correlated with altered host plant gene regulation and the *Pht1;6* gene is thus shown to be an important component of the genetic architecture underpinning the complex trait, AM symbiosis.

## Results and discussion

### Phosphate transporter gene *Pht1;6* is disrupted in maize *mutator* mutant *pht1;6*

**Figure 1:**
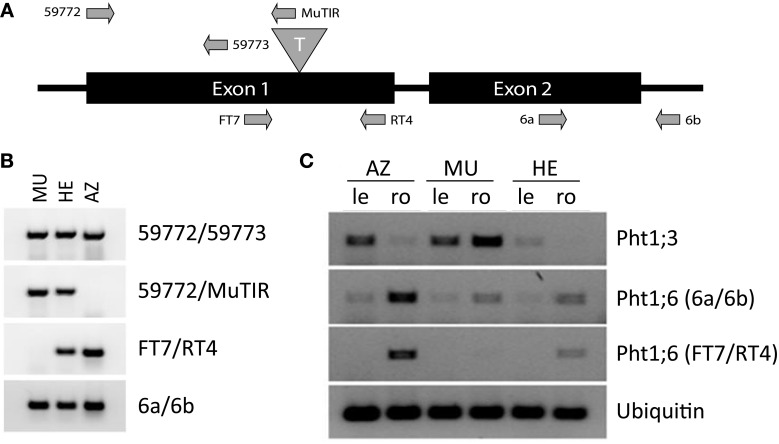
**Molecular genetic analysis of *Mutator* element insertion in *Pht1;6.* (A)** Schematic representation (not to scale) of the *Pht1;6* gene, transposon (T) insertion site and location of sequences to which primers (arrows) used for PCR experiments anneal (primers were subsequently used in **(B,C)**. **(B)** Detection of mutant and wild type allele, respectively, of *Pht1;6* in leaf genomic DNA of segregants of 2nd backcross. **(C)** Detection of *Pht1;3* and *Pht1;6* transcripts, respectively, via RT-PCR. RNA was isolated from MU, HE, and AZ mature leaves (le) and mycorrhizal roots (ro) of segregants of 2nd backcross taken from the -P [+NK] field. *Pht1;6* Primers FT7/RT4 are transposon flanking primers, while 6a/6b are 3' downstream of the transposon insertion site of the *Pht1;6* gene. *Ubiquitin* transcripts were used as cDNA normalization control.

A reverse genetics analysis employing the Trait Utility System for Corn (TUSC) (McCarty and Meeley, 2009) revealed F1 plants carrying an insertion of the *Mutator (Mu)* transposable element in the first exon, 855 bp downstream of the initiation codon ATG of the *Pht1;6* gene generating the mutation *pht1;6::Mu* (Figure 1A). Heterozygous (HE) plants were self pollinated and progeny was used to investigate segregation of *pht1;6::Mu* (Table S1). To determine the genotype of the plants utilized for greenhouse and agricultural field experiments, PCR with genomic DNA and specific primer pairs for mutant (59772; MuTIR) and wild type (RT4; FT7) *Pht1;6* alleles, respectively (Figure 1B and Table S2) was applied. Mutant and wild type genotype was determined from leaf samples as plants grew. Segregation analysis suggested Mendelian inheritance of the *pht1;6::Mu* mutation under the assumption that survival of homozygous *pht1;6* plants (MU) was reduced under field conditions (Table S1; our own observation). The mutation was stably transmitted to the F2 generation as well as throughout the subsequent series of backcrosses to the maize inbred line B73.

To monitor *Pht1;6* transcript levels seeds, segregating for the Mu insertion into the *Pht1;6* gene from 2nd back-cross to inbred B73 maize followed by self-pollination were used. These seeds were taken from a single cob and sown on agricultural lots differing in their P availability located at the Agroscope-Reckenholz-Tänikon (ART) Research Station in the vicinity of Zurich (Switzerland). At ART, lots with 8-year crop rotation have been fertilized since 1987 with nitrogen and potassium but omitting P (−[P] +[NK]) or were not fertilized at all (−[PNK]), while control lots were fully fertilized (+[PNK]). Soil of −[P] +[NK] and −[PNK] lots was depleted of P in comparison to +[PNK] lots (Table S3). A semi-quantitative PCR assay, using primers flanking the transposon insertion (Figures 1C, S1), indicated the absence of full-length *Pht1;6* transcripts in roots and leaves of MU plants. In HE and azygous (AZ, homozygous for the undisrupted *Pht1;6* gene, i.e., all wild type descendants) siblings, full length *Pht1;6* mRNA accumulated in mycorrhizal roots which was in agreement with previous work (Nagy et al., 2006). Levels of transcripts of the direct Pi uptake pathway gene *ZEAma;Pht1;3* (*Pht1;3*) (Glassop et al., 2005; Nagy et al., 2006) were highest in roots of field grown MU plants (−[P] +[NK]), indicating that the roots suffered from Pi starvation. Differences in *Pht1;6* expression between AZ and HE plants relative to *Ubiquitin* could be due to a lower rate of arbuscular root colonization (year 2006, not shown) or point toward haploinsufficiency (compensated by a single wild-type allele) in heterozygous plants. In contrast to humans and yeast, few cases of haploinsufficiency have been documented in *Arabidopsis*; a single functional allele is therefore sufficient for most cellular functions (Meinke, 2013). Thus, in the following series of field trials, we carefully investigated phenotypical differences among MU, HE, and AZ plants. However, HE and AZ were alike which indicated that reduced *Pht1;6* expression in HE allowed adequate Pi uptake under field conditions.

The *pht1;6::Mu* mutation is in the inbred line B73 background. B73 is the most widely used inbred line of maize and it is the common parent shared by all lines of the maize nested association mapping population (Yu et al., 2008). The line had been sequenced to high quality using a map-based clone-by-clone strategy (Schnable et al., 2009). In spite of its high relevance for research and breeding, line B73 is not readily transformable. Thus, functional complementation of *pht1;6* by genetic transformation with the wild type Pht1;6 allele is currently not feasible. Moreover, despite our continuous efforts it was not possible to obtain a second *pht1;6* mutant in any of the available public databases. On the other hand, phylogenetic analysis clearly substantiated a single *Pht1;6* ortholog in the maize genome, and also backcross 5 (not shown) exhibited a robust *pht1;6* mycorrhizal phenotype described below indicating that the lesion that causes the phenotype maps to within some very close distance to the *Mu* insertion in the *pht1;6* allele. Our experiments with *pht1;6* provided robust phenotypical and physiological data which delivered correlative evidence for Pht1;6 function under controlled and agricultural conditions. The results are consistent with previous work in rice which established an important role for the rice ortholog ORYsa;PHT1;11 (OsPT11) in MPU (Paszkowski et al., 2002; Yang et al., 2012).

### Disruption of *Pht1;6* correlates with yield losses of maize under P-limited conditions in the field

To investigate the possible impact of the mutation on plant performance in agricultural field conditions, maize seeds segregating for *pht1;6::Mu* from a single cob were sown on agricultural lots located at the ART-Research Station. For the trials in 2006, 2007, and 2009, seeds that originated from 2nd, 3rd, and 4th back-cross to inbred B73 maize followed by self-pollination were used.

**Figure 2:**
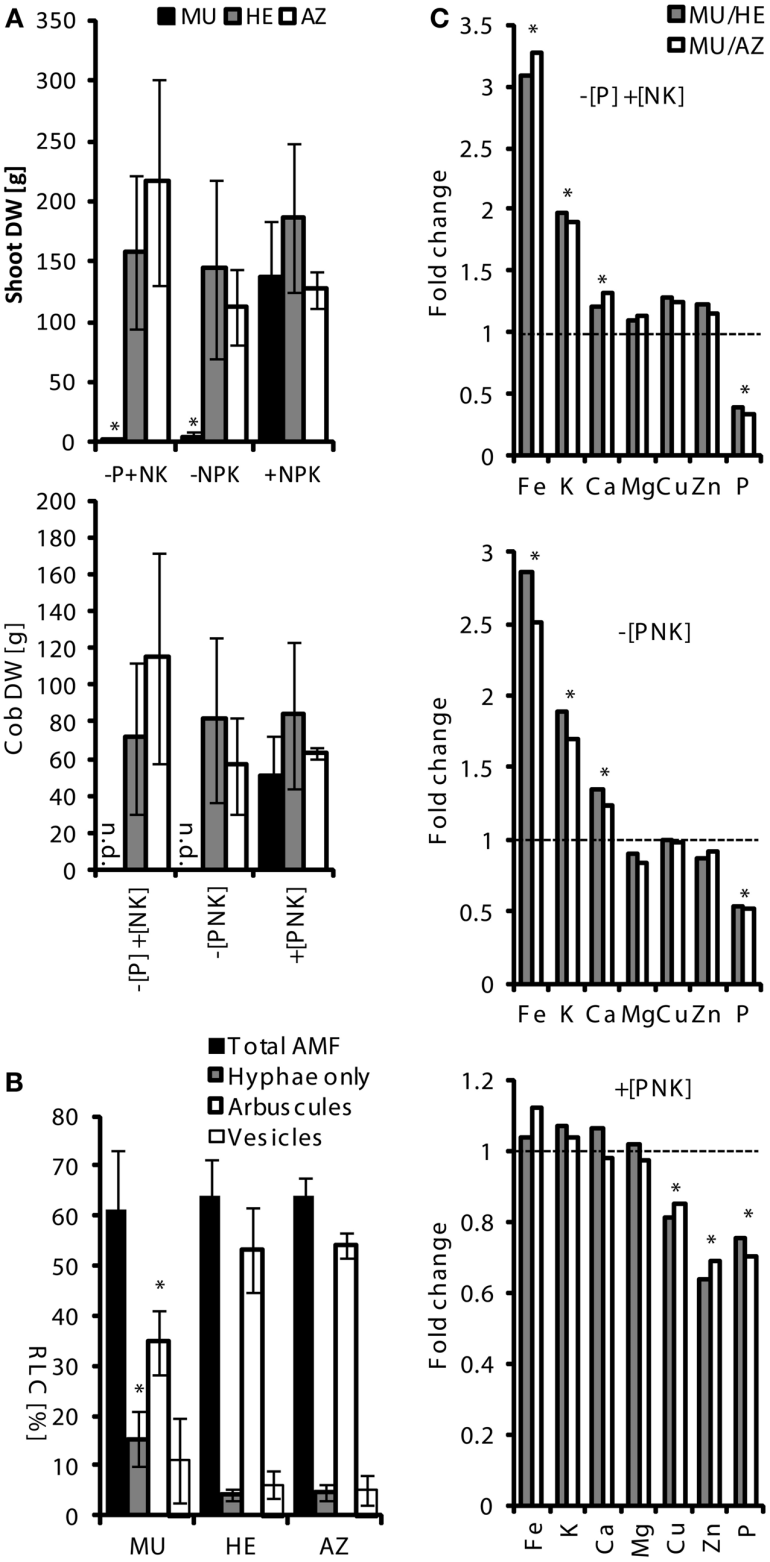
**Phenotype of *pht1;6* mutant maize as a function of soil P status in the field.** For field trials, maize seeds were produced by several back-crosses of plants carrying the *pht1;6::Mu* mutation to B73. After each backcross, self-pollination was done in order to obtain a seed population with segregation of the transposon insertion. Segregating seeds from a single cob of the 4th backcross were sown in spring 2009 and grown for a vegetation period (about 6 months) in parallel on fields that, since 1989, had been fertilized with the macronutrients N, P, and K (+[PNK]), N,K without P (−[P] +[NK]) or without N,P and K (−[PNK]). Status of zygosity toward *pht1;6::Mu* (MU = homozygous; HE = heterozygous; AZ = azygous) was verified using PCR on genomic DNA of leaves (Table S2). Number of samples (*n*): *n*−[P] +[NK] = 6.12/8 (MU/HE/AZ); *n*−[PNK] = 4/11/10; *n*+NPK = 7/16/4. **(A)** Dry weight (mean ± *SD*) of shoot (upper panel) and cobs (lower panel) of *pht1;6::Mu* segregants grown under the indicated field conditions are shown. Significant differences of mean values within one plant genotype were determined by One-Way ANOVA analysis (^*^p < 0.05) and are indicated by asterisks. n.d., not detected. **(B)** Percentage of root length (means ± *SD*) of total and arbuscular colonization of roots (RLC) from −[P] +[NK] fields. Significant differences of mean values between genotypes within corresponding mycorrhizal categories were determined by One-Way ANOVA analysis (^*^p < 0.05) and are indicated by asterisks. **(C)** Mean values of element concentrations of MU shoots divided by the mean values of HE (MU/HE) or AZ (MU/AZ) shoots indicate magnitude of change of MU vs. HE or AZ, respectively. Dashed line at *y* = 1 means “no fold change”. Significant differences of mean values used for calculating the ratio were determined by One-Way ANOVA analysis (^*^p < 0.05) and are indicated by asterisks. n.d., not detected.

Segregating seeds (mixture of MU, HE, and AZ plants) from a single cob were sown in spring 2006, 2007, and 2009 and grown for a vegetation period (about 6 months) in parallel on the fields (+[PNK]); −[P] +[NK] or −[PNK]). Status of zygosity toward *pht1;6::Mu* was verified using PCR on genomic DNA of leaves (as described above, Table S2). Phenotypic differences between field-grown plants severely occurred in low P soils (−[P] +[NK] and −[PNK]), where shoot length (not shown), cob development and hence grain yield, as well as biomass of MU plants were strongly reduced (Figures 2A, S3). In contrast, P supply in +[PNK] treatments restored the growth phenotype including cob development of MU plants. HE and AZ plants produced comparable levels of shoot biomass in all three lots. Despite great phenotypic differences root colonization by AMF in field grown MU plants did not differ substantially from those in HE and AZ roots. Moreover, exploration of AM fungal diversity by Sanger sequencing (Sanger et al., 1977) of ITS failed to show structural differences in the AM fungal assemblages in these roots (data not shown).Our field studies suggested a high dependence of maize productivity on MPU pathway activity in agricultural soils at low P fertility levels.

Total root colonization by AMF in field grown MU plants was about 60% and did not differ substantially from those in HE and AZ roots (see above). Root systems of neighboring plants overlapped and presumably MU roots were colonized from neighboring HE and AZ plants. On a structural level, however, arbuscular colonization in MU roots was lower while colonization of root sectors with hyphae was significantly higher in P depleted (−[P] +[NK]) soil (Figure 2B).

### Elemental composition of *Pht1;6* mutant is affected under P-limited field conditions

Next, a possible contribution of MPU activity to the overall nutrient status of the host plant was investigated. To this end, we performed multi-elemental fingerprinting on maize shoots using inductively coupled plasma mass spectrometry (ICP-MS) to determine the concentrations of nutrient elements. We were interested in learning whether elemental fingerprints are indicative of a functional MPU pathway under field conditions. Total P concentration in aerial tissues of MU as compared to HE and AZ plants was significantly lower in all field lots (Figures 2C, S3) which is indicative by a fold change smaller 1. The difference was most pronounced under P depletion conditions in (−[P] +[NK] and −[PNK]) soils. Under P limited conditions, concentrations of magnesium, copper and zinc remained largely unchanged in MU shoots, while iron, potassium and calcium levels were enhanced compared to HE and AZ plants. Increased iron content during Pi starvation has previously been reported for Pi starved *Arabidopsis thaliana* and rice in liquid cultures (Hirsch et al., 2006; Ward et al., 2008; Zheng et al., 2009) and *Arabidopsis* and maize pot cultures (Baxter et al., 2008; Schluter et al., 2013).

At −[P] +[NK] conditions, MU plants contained enhanced copper and zinc levels in addition to reduced P and enhanced Fe levels in comparison with MU plants at +[NPK] fields (Table S4). These results are in agreement with the characteristic elemental fingerprint shown for Pi deficiency by Baxter et al. (2008) in *Arabidopsis* leaves. In contrast, mycorrhizal HE and AZ plants grown on −[P] +[NK] fields compared to +[NPK] fields (Table S4) did not show a clear Pi deficiency-related elemental fingerprint and no significant differences in biomass or cob yield (Figure 2A) which suggested that a functional MPU pathway at least in part compensated for low Pi availability under agricultural field conditions. Interestingly, the lower P concentration in MU plants grown on +[PNK] was not reflected in a significant decrease in biomass (Figure 2A). Similarly P concentrations in cobs of MU from +[NPK] plants were similar to those in cobs of HE and AZ plants (Figure S4). This indicated that sufficient Pi could be allocated to developing cobs and P was not limiting growth under +[NPK] field conditions in a genotype like *pht1;6* exhibiting impaired MPU activity. Overall, this data is in line with published work (see for example Jakobsen et al., 2001; Smith et al., 2003, 2004) and indicates that the MPU pathway significantly affected mineral allocation to the shoot under Pi limiting field conditions. It could be interesting to study, whether a specific multi-elemental fingerprint, such as e.g., low Fe/low K/high P (see Figure S3), could be a useful marker for the breeding of P uptake efficient mycorrhizal maize for agricultural production in low P fields.

### Reduced mycorrhizal colonization level in *Pht1;6* mutants can be restored by *trans*-complementation

**Figure 3:**
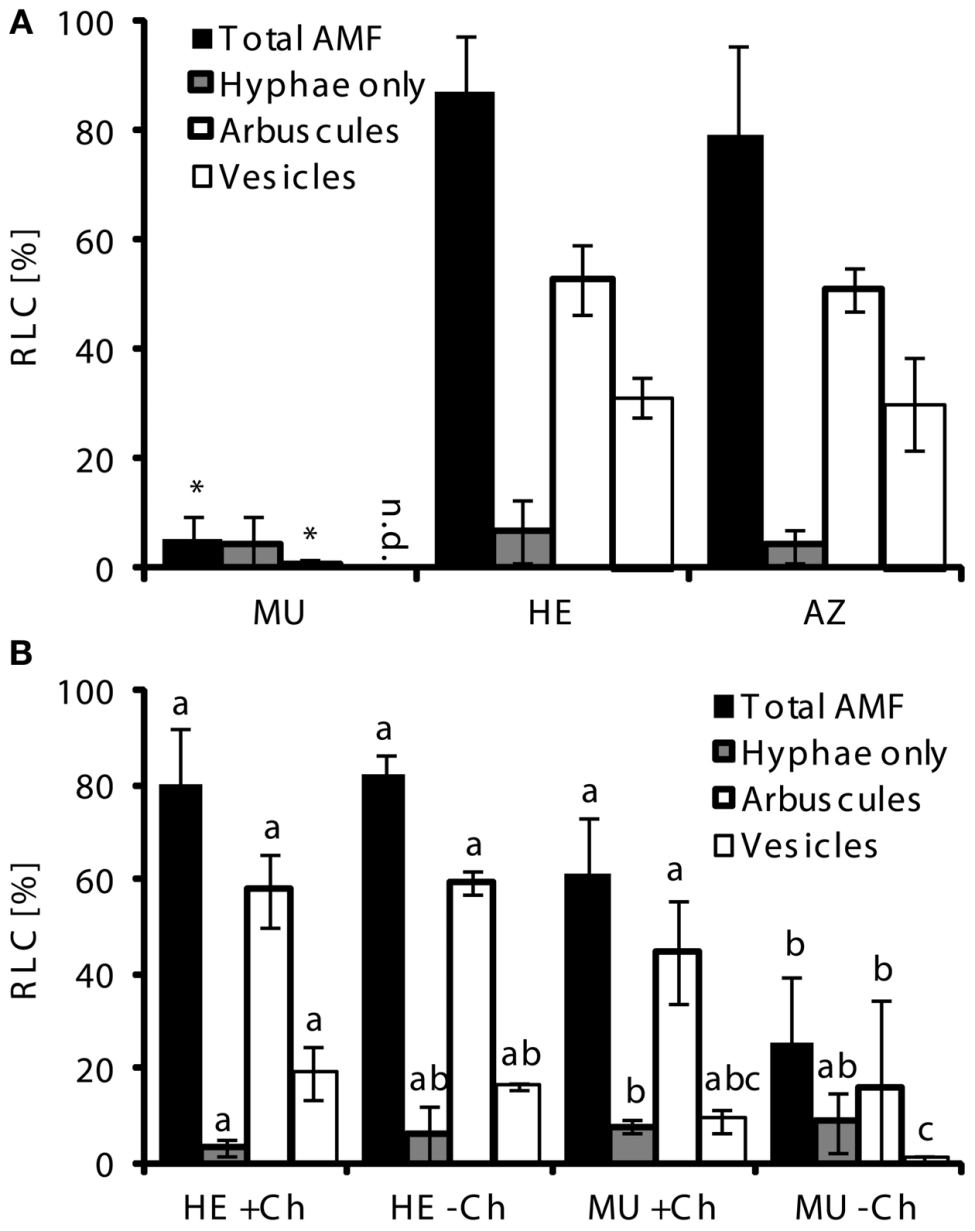
**AMF colonization in mutants depends on presence of nurse plants. (A)** Root length colonized (RLC) by AMF of MU, HE, and AZ root samples (each *n* = 5) of *pht1;6::Mu* segregants of 3rd backcross grown under greenhouse conditions in pots inoculated with *Glomus intraradices* (syn: *Rhizophagus irregularis*) BEG 75 and in absence of nurse plants. **(B)** RLC by AMF of MU and HE root samples (each *n* = 3) derived from 4th backcross grown in pots together with mycorrhizal (*G. intraradices*, BEG75) chive nurse plants. Means and standard deviations of total, arbuscular and vesicular colonization in percentage of root-length by AMF are shown. Significant differences of mean values between genotypes and treatments within groups of fungal structures were determined by One-Way ANOVA analysis (*p* < 0.05) and are indicated by either asterisks or letters. Means and bars with the same letter are not significantly different. n.d., not detected.

We tested the possibility if mycorrhizal MU plants, when grown alone or mixed with other species under controlled conditions, would develop like MU in the field. In a conventional greenhouse, in contrast to the field, mycorrhizal colonization rates differed greatly among the three genotypes 9 weeks after inoculation with *Glomus intraradices* (syn: *Rhizophagus irregularis*) (Raab et al., 2005; Redecker et al., 2013), BEG75 (Figure 3A). Colonization of roots of both AZ and HE averaged 80–87%, while the fungus was unable to penetrate and proliferate in the roots of *pht1;6*, i.e., AM symbiosis was poorly developed in MU with only about 5% of root length colonized. This stood in agreement with Yang et al. (2012) who showed that AM fungal root colonization rates were strongly reduced in pot-grown rice mutants carrying loss-of-function mutations in the Pi transporters OsPT11 and ORYsa;PHT1;13 (OsPT13) when compared to wild type roots. As the mycorrhizal phenotype in HE was similar to that in AZ in all our experiments conducted so far, HE was used as control for MU in subsequent experiments.

**Figure 4:**
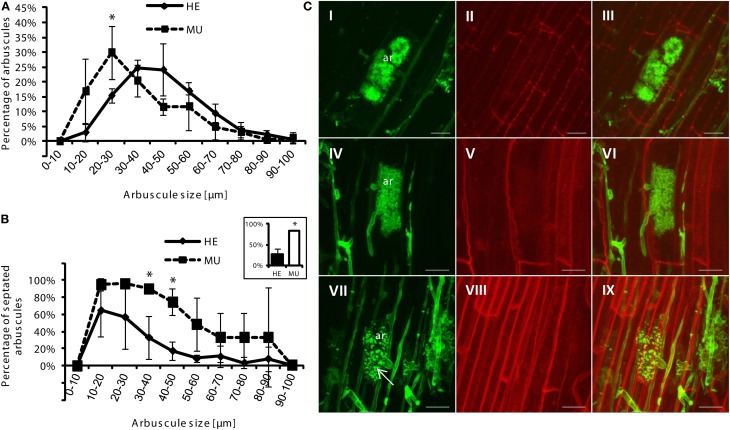
**Analysis of arbuscule size and septation in roots of HE vs. MU plants.** Roots were taken from the ^33^P uptake studies at the end of the experiment. Means and standard deviations of **(A)** arbuscule size classes, and **(B)** percentage of septated arbuscules of different sizes classes in roots of HE and MU plants are shown. Inset shows total percentage of septation. Arbuscule sizes and percentages of septation in 150 root sections in HE and MU roots (each *n* = 3) were quantified. Significant differences for *p* < 0.05 (student's *t*-test) are indicated by asterisks. **(C)** Confocal microscopy shows characteristic images of arbuscules (ar) of HE **(I–III)** and MU **(IV–IX)** plants. Images of fungal cell walls stained with WGA-Alexa Fluor 488 **(I, IV,** and **VII)**, autofluorescence of plant cell walls **(II, V,** and **VIII)** and corresponding overlays **(II, VI, IX)** are shown. Scale bars = 20 μm.

To examine whether plants that are already well colonized by AMF can stimulate mycorrhiza formation in neighboring pot-grown MU plants via *trans*-complementation, we used mycorrhizal *Allium schoenoprasum* L. (chive) as nurse plants during 9 weeks of co-culture with HE or MU. This should mimic the situation of individual MU plants derived from segregating seeds in the field which grew close to AZ and HE plants. Total AMF colonization in roots of HE plants was about 80%, independent of the presence of a nurse plant (Figure 3B). In MU plants grown for 9 weeks in presence of mycorrhizal chive, root length colonized by AMF was almost as high as in HE plants. In contrast, when chive shoots were excised 5 weeks before the end of co-culture, *pht1;6* mutants exhibited a significantly reduced colonization phenotype (26% colonization). *Trans*-complementation was also observed in *M. truncatula* mutants defective in Pi transporter MtPT4 activity (Javot et al., 2011). Thus, this suggested that *trans*-complementation is dependent on shoots of neighboring mycorrhizal plants which are likely to equip the fungus with photosynthetically fixed carbon to allow completion of its life cycle in MU roots. It is therefore conceivable that under the field conditions described above *trans*-complementation allowed efficient colonization of *pht1;6* mutants despite strongly limiting MPU in these plants. This would corroborate recently provided physiological data (Kiers et al., 2011) emphasizing the effect of P availability on C allocation to the AMF, although in that work Pi transporter function had not been investigated. MU alone can hardly sustain AM symbiosis because total root length colonization is reduced >15-fold compared to HE (see Figure 3A) and the percentage of senescing arbuscules is increased by almost 4-fold (see Figure 4 discussed below). Still normal arbuscules were present in MU roots. It will be interesting to study which nutrient transport processes still occur in these presumably intact arbuscules.

### Uptake of radioactively labeled ^33^P-Pi is non-functional in *Pht1;6* mutants

**Figure 5:**
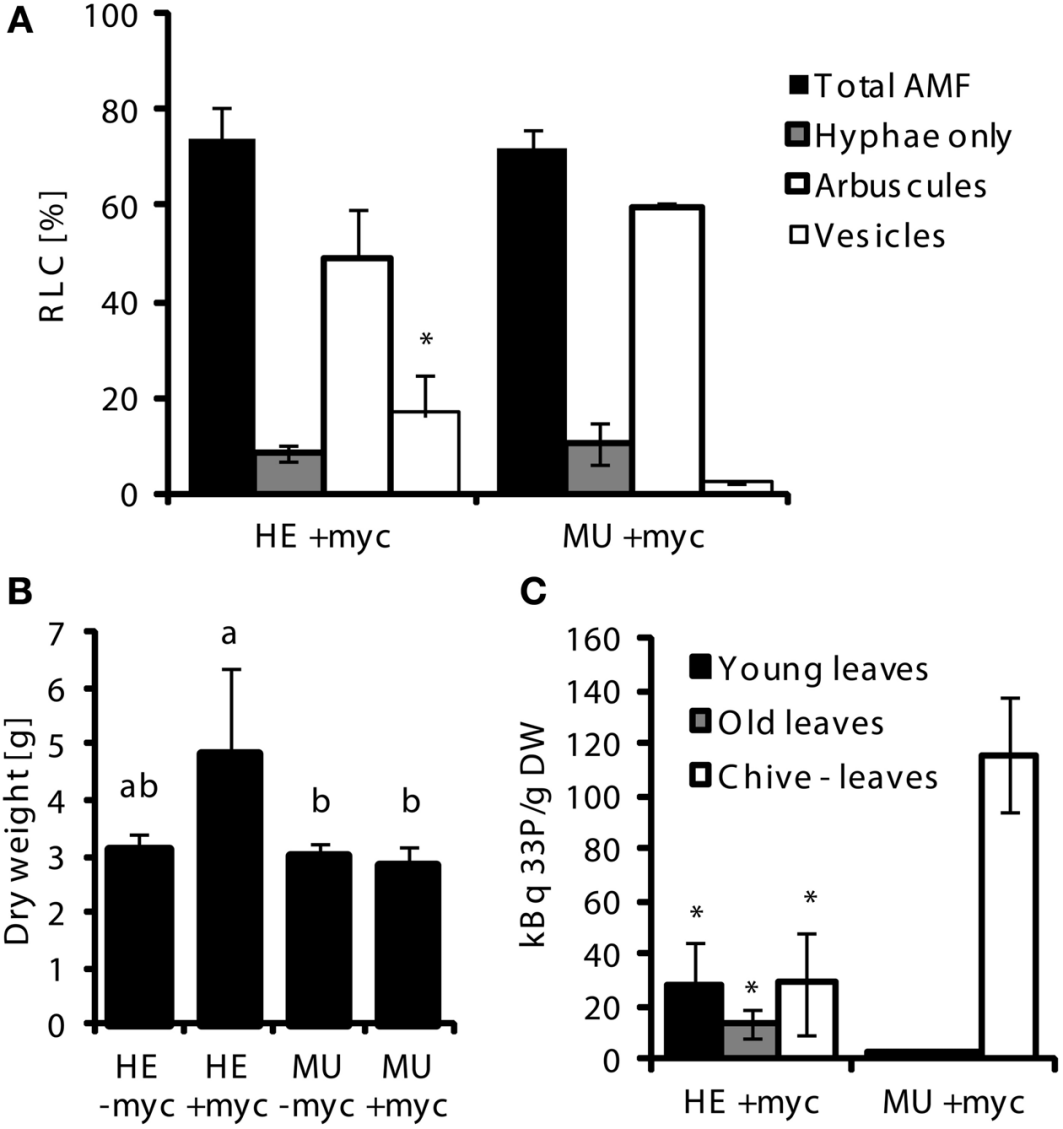
**Uptake of ^33^P-labeled orthophosphate (Pi) via mycorrhizal Pi uptake pathway.** For Pi uptake studies, single MU or HE plants derived from 4th backcross were grown in pots together with a mycorrhizal (*G.intraradices*, BEG75) chive nurse plants (+myc) or chive without AMF (-myc) for 8 weeks at low Pi conditions (*n* = 3). Means and standard deviations of **(A)** root length colonized (RLC) by AMF of MU and HE root samples taken after 5 weeks and 5 days, 2 days before injection of labeled Pi into the hyphal compartment (HC); **(B)** shoot dry weight at harvest; and **(C)** ^33^P signals detected in aerial tissues of MU and HE maize plants 2 weeks after injection of labeled Pi into HC are shown. Significant differences of mean values between genotypes and treatments within groups of fungal structures were determined by One-Way ANOVA analysis (*p* < 0.05) and are indicated by either asterisks or letters. Means and bars with the same letter are not significantly different.

Next, we assessed the contribution of the MPU pathway to total P uptake in mycorrhizal maize plants by delivering ^33^P-Pi via AMF hyphae in a bi-compartmented cultivation system (see “Material and Methods”). Here we again used mycorrhizal chive nurse plants neighboring maize at low Pi conditions to allow sufficient colonization of MU. Lengths of roots colonized by AMF 2 days before injection of radiolabeled P into the HC did not differ between HE and MU plants, except for vesicular colonization that was clearly increased in HE plants (Figure 5A). At this time point of balanced colonization in HE and MU plants, ^33^P-Pi was added to the hyphal compartment and 2 weeks after the addition of labeled Pi plants were harvested. MU and HE mycorrhizae from this experiment were used for staining of fungal cell walls with WGA Alexa Fluor and arbuscule sizes were quantified (Figure 4). The abundance of arbuscule size classes was largely similar in MU and HE plants with a shift toward smaller arbuscules in MU roots (Figure 4A). The latter was previously observed in the studies of Javot et al. (2007) and Yang et al. (2012) in *Medicago* and rice, respectively. Arbuscules of two size classes (30–40 μm and 40–50 μm) in MU roots showed a higher rate of septation which suggests a faster turnover of the symbiotic structures with premature senescence (Figure 4B). Nonetheless there was a considerable amount of mature non-septated arbuscules in MU roots that were comparable to those in HE roots (Figures 4B,C). In accordance with this and with results from the monocot rice indicating the presence of live arbuscules in mutants of OsPT11 and OsPT13 that are described as essential for a functional MPU pathway (Paszkowski et al., 2002; Yang et al., 2012), nutrient exchange via those arbuscules might still occur. To address this question, allocation of radiolabeled P to the shoot and shoot growth were analyzed. Mycorrhizal HE plants accumulated significantly more biomass than mycorrhizal MU plants (Figure 5B), while the root/shoot ratio remained unchanged (not shown). The leaves of HE plants accumulated 6–10-fold higher ^33^P levels than MU plants (13–28 kBq ^33^P/g dry weight (DW) in HE vs. ~2 kBq ^33^P/g DW in MU) (Figure 5C). Leaves of chive in co-culture with HE plants accumulated ^33^P at levels similar to those in HE maize leaves (~28 kBq ^33^P/g DW), while chive co-cultured with MU plants accumulated significantly more radiolabel P in leaves (~115 kBq ^33^P/g DW). No ^33^P was detectable in leaves from non-mycorrhizal samples indicating that no Pi had diffused into the root compartment and suggesting that Pht1;6 is responsible for Pi uptake via MPU. These results with maize Pht1;6 facilitating MPU are well in agreement with the above described studies on the orthologous Pi transporter OsPT11. Moreover, our work extends the model of Merrild et al. (2013) by showing that interspecific and size-asymmetric competition between maize and chive is mainly determined by mycorrhizal Pi transport activity in the maize plant linked into the same CMN as chive. Here we compared ^33^P in maize and in chive leaves in the two situations in which either HE or MU is the plant neighboring the chive plant. As shown in Figure 5C ^33^P concentrations in leaves of chive and neighboring HE plants were similar but chive leaves accumulated 60-fold higher ^33^P concentrations as leaves of neighboring MU maize. This showed that loss of Pht1;6 activity in MU stimulated Pi allocation to the smaller species chive. The use of plant mutant genotypes like pht1;6 varying in mycorrhizal Pi transport activity could prove useful in ecological genetic studies on Pi fluxes in mixed plant populations and plant communities on soils differing in Pi availability or subject to different agricultural management.

Besides of mycorrhizal roots, *Pht1;6* is expressed in maize leaves under Pi-deficient conditions (Nagy et al., 2006; Colmsee et al., 2012). In our study, *Pht1;6* transcript abundance was close to the detection limit in leaf tissue (Figures 1C, S1). To evaluate a putative additional function of Pht1;6 in Pi transport in the shoot, translocation studies were carried out using radioactively labeled ^33^P which was added to source leaves or roots (Figure S5). The results revealed no significant differences in the allocation of Pi to different leaves between MU plants, HE, and AZ, respectively. In accordance with this finding, Pi uptake via phloem and phloem unloading/transport within leaf tissue was not affected by a Pht1;6 loss-of-function. Moreover Pi uptake via roots and root/shoot transport was not affected by the mutation. This may be due to functional redundancy in Pi transporter-mediated ^33^P distribution. Overall, we believe that it is unlikely that the physiological phenotypes in mycorrhizal MU, which were predominantly observed during Pi deficiency, were the result of altered Pi allocation at the level of the shoot. Therefore, we propose that these physiological and structural differences in MU compared with the controls are a consequence of a loss-of-function mutation in *Pht1;6* which strongly affects mycorrhiza functioning.

### Transcriptome analysis suggests differences in gene expression between control and MU plants

**Figure 6:**
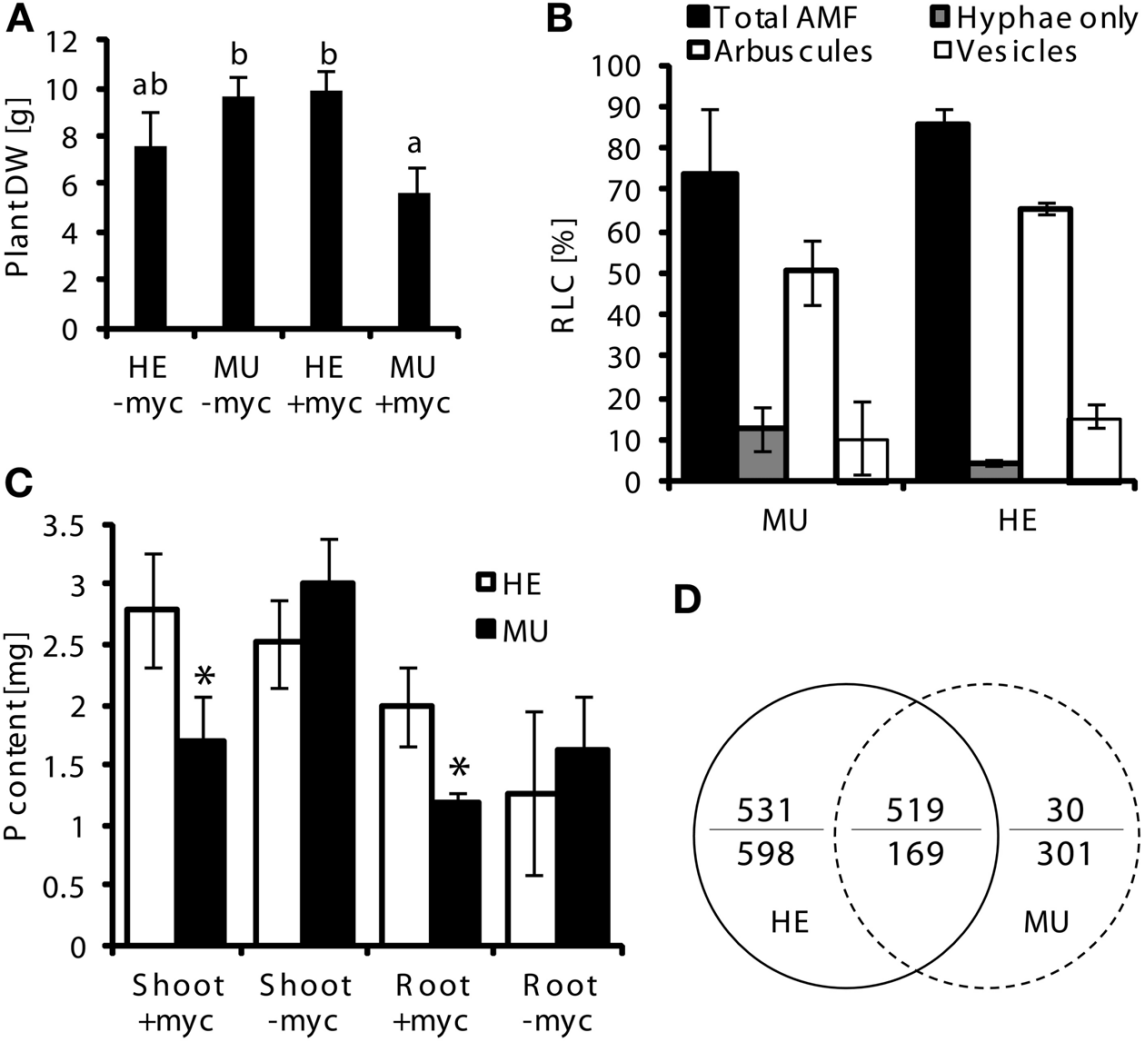
**Assay for trancriptomics in mycorrhizal roots of *pht1;6* mutants and heterozygous plants.** MU and HE plants (each *n* = 3) derived from 4th backcross were grown in single pots together with mycorrhizal (*G. intraradices*, BEG75) chive nurse plants (+myc) or chive without AMF (-myc) for 8 weeks at low Pi conditions. Means and standard deviations of **(A)** dry weight of total plants, **(B)** root length colonized (RLC) by AMF of MU and HE root samples and **(C)** P content detected in shoots and roots of MU and HE maize plants are shown. Significant differences of mean values between genotypes and treatments within groups of fungal structures were determined by One-Way ANOVA analysis (*p* < 0.05) and are indicated by either asterisks or letters. Means and bars with the same letter or without a label are not significantly different. **(D)** Venn statistic of significantly (corrected *p* < 0.01) mycorrhiza regulated genes in MU (right circle) and HE (left circle) roots and co-regulated genes (overlap). Number above the line indicates up regulated genes, numbers below the line indicates down regulated genes.

**Figure 7:**
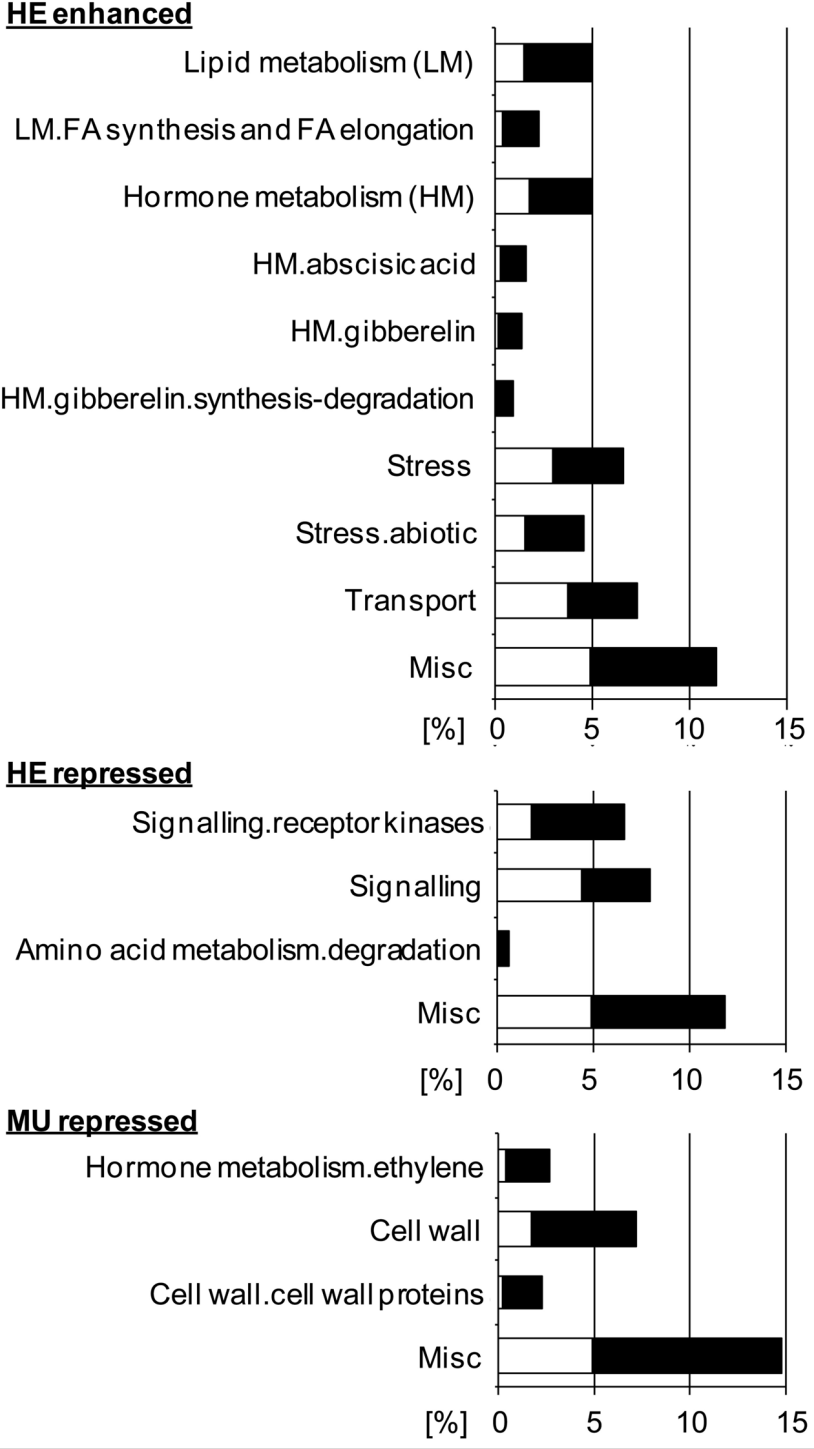
**Gene set enrichment analyses of genes regulated upon mycorrhization in MU and HE roots (each *n* = 3).** Analysis was performed using “MAPMAN.” White area indicates expected gene set; black area indicates overrepresentation of gene set in percent. Only overrepresented gene sets are shown.

To investigate whether loss of Pht1;6 activity affects gene expression of roots which are colonized with AM fungi, transcriptome sequencing of mycorrhizal and non-mycorrhizal MU and HE roots was performed through paired-end mRNA sequencing on the Illumina Genome Analyzer II platform. MU and HE plants were grown in the presence of either mycorrhizal or non-mycorrhizal chive plants under low Pi conditions during 8 weeks. At harvest mycorrhizal MU plants grew like non-mycorrhizal HE but showed significantly less biomass than mycorrhizal HE and non-mycorrhizal plants, respectively (Figure 6A). Root/shoot ration, root length colonized and rate of mycorrhizal structures did not differ among HE and MU plants which allowed comparative RNA sequencing using material of similar mycorrhizal status (Figure 6B). A significantly higher P content in shoot and roots of mycorrhizal HE compared to MU plants was apparent (Figure 6C) but in contrast to P content, concentrations of P in the tissues did not differ (Figure S6). This finding alludes to an increased Pi uptake via MPU pathway in HE compared to MU plants that contributes to significant biomass accumulation while cellular P concentration was maintained. Comparative transcriptomics with MU and HE roots (ratios between mycorrhizal and non-mycorrhizal roots, indicated by +myc/-myc) revealed genotype-specific gene regulation. In mycorrhizal vs. non-mycorrhizal HE plants up- and down-regulation of 531 and 598 genes, respectively, occurred (Figure 6D and Data S1). In contrast, in case of MU plants expression of only 30 genes was specifically enhanced in mycorrhizal roots, while 301 genes were repressed (Figure 6D and Data S1). Gene set enrichment analyses (Figure 7) revealed in mycorrhizal HE but not in MU plants an enhanced expression of genes involved in lipid and hormone metabolism (especially gibberellin and abscisic acid metabolism). On the other hand, transcript levels of signaling genes, especially receptor kinases, and genes involved in amino acid metabolism (e.g., aspartate degradation) were repressed in HE. In MU plants, over-represented repressed genes were involved in cell-wall synthesis and ethylene metabolism. The low number of genes exhibiting enhanced expression in MU did not allow gene set enrichment analysis. Within the category “cell wall” transcripts of two genes that are homologous to α(1,2)fucosyltransferase (FUT) genes are repressed between log2 and 4-fold in MU roots. In *Arabidopsis* FUTs are involved in cell wall biosynthesis and responsible for the glycosylation of arabinogalactan proteins (Wu et al., 2010).

Within the subcategory “cell wall.cell wall proteins” several genes homologous to sequences coding for Leucine-rich repeat (LRR)/ leucine-rich repeat/extensin (LRX), expansin or FLA (fasciclin-like arabinogalactan) proteins were repressed between log1.5 and 2.4-fold specifically in MU plants. LRR/LRX proteins are localized in the cell wall and regulate root hair morphogenesis in *Arabidopsis* (Baumberger et al., 2001; Velasquez et al., 2011). Moreover, expression of extensin-like genes is confined to tip growth in tomato root hair cells and pollen tubes (Bucher et al., 2002). Also there is evidence that expansins are required for root hair tip growth (Lin et al., 2011). Biological functions of the various higher-plant FLAs are not clear, but are reported to be closely linked to secondary cell wall biosynthesis (Seifert and Roberts, 2007). Taken together, our analyses suggest a considerable impact of Pht1;6 activity on the regulation of cell wall biosynthesis in AM.

In category “hormone metabolism.ethylene” (Figure 7) mainly genes that are homologous to AtERF4, 7, 11, and 12 are repressed between log2 and 4.5-fold in MU roots. Yang et al. (2005) demonstrated that AtERF4, AtERF7, AtERF11 and AtERF12 can function as transcriptional repressors. Moreover, *Arabidopsis* ERF4 is capable of modulating ethylene and abscisic acid responses. In addition AtERF4 was suggested to act as a novel negative regulator of JA-responsive defence gene expression and resistance to the necrotrophic fungal pathogen *Fusarium oxysporum*. Our results therefore suggest that specific ERF transcription factors modulate hormone metabolism and possibly enhance plant defence mechanisms in roots of mycorrhizal *pht1;6* mutants (McGrath et al., 2005).

### Pi transporter gene expression in maize mycorrhizae

**Figure 8:**
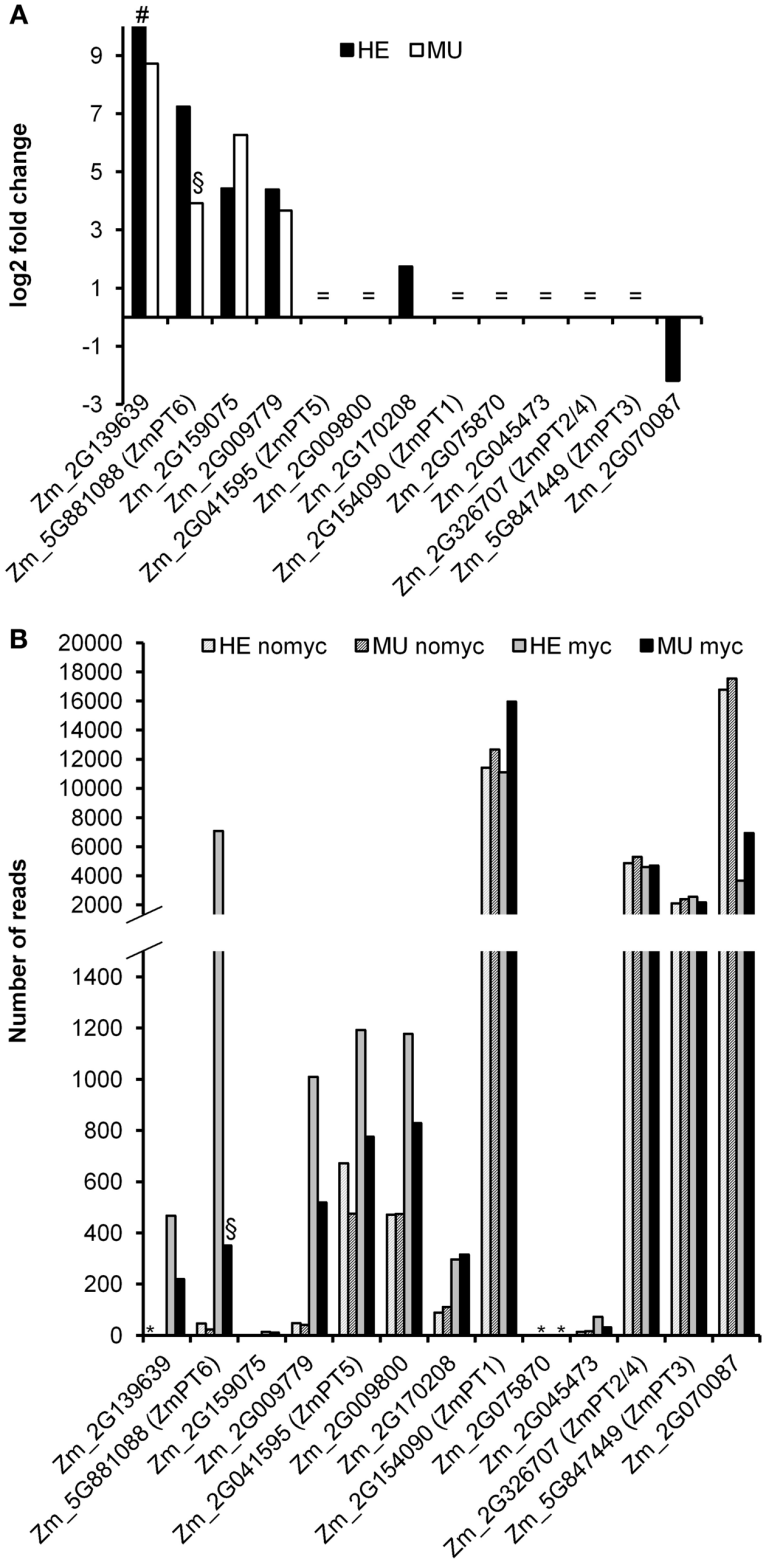
**Transcript levels of maize Pht1 genes. (A)** Log2 fold changes of Pht1 gene transcripts comparing mycorrhizal vs. non-mycorrhizal roots of the genotypes MU and HE (each *n* = 3). = shows that genes are expressed but not regulated in mycorrhizal vs. non-mycorrhizal conditions; # indicates a gene that is exclusively expressed in mycorrhizal conditions. **(B)** Expression (number of reads) of the *Pht1* genes within the experimental conditions. Lack of expression is indicated by an asterisk (^*^). Fold change of *pht1;6* mutants (§) is due to false positive annotation of transcript-fragments identical to *Pht1* gene homologs or to *Pht1;6* transcripts 3′ upstream and 5′ downstream of the transposon insertion site. Transcripts spanning this insertion site were absent (see Figure S7).

Within each genotype the expression of the Pi transporter genes *Pht1;2/4* and *Pht1;3* remained unchanged in mycorrhizal compared to non-mycorrhizal plants (Figure 8). This reflected equal P concentrations observed in these plants (Figure S6). The same *Pht1* genes were previously shown to be differentially regulated upon Pi starvation (Glassop et al., 2005; Nagy et al., 2006). Mapping of the short RNA reads to the maize B73 *Pht1* gene sequences revealed expression of overall 13 *Pht1* genes in HE and MU plants. In MU plants transcripts spanning the *Mu* insertion site were absent (Figure S7), but false positive annotations of fragments identical to *Pht1* homologs or to *Pht1;6* transcripts 3′ upstream and 5′ downstream of the insertion site could be observed. The absolute RNA read counts revealed a set of five highly abundant Pht1 gene transcripts (Pht1;1, 2/4, 3, 6, and Zm_2G070087 and a set of eight lowly expressed genes (Pht1;5, Zm_2G009800, Zm_2G009779, Zm_2G139639, Zm_2G170208, Zm_2G045473, Zm_2G159075, and Zm_2G075870; for maize *Pht1* gene nomenclature see Yang et al., 2012), whereas the read number for Zm_2G159075 is one of the lowest. Among all 13 genes, four were strongly induced upon mycorrhization, i.e., *Pht1;6*, *Zm_2G009779*, *Zm_2G139639*, and *Zm_2G159075* with a log2 fold change >4. Interestingly the *Pht1* gene *Zm_2G159075* was strongly up regulated in mycorrhizal MU compared to mycorrhizal HE roots. Except for *Pht1;6*, transcript levels of the other mycorrhiza-regulated *Pht1* genes were similar in mutant and controls. Phylogenetic analysis of the Pht1 protein sequences from maize, rice, and other plant species using non-parametric bootstrapping (Yang et al., 2012) resulted in a consensus tree where Zm_2G159075 clustered with OsPT13. The radiotracer uptake studies suggested that an increase in *Zm_2G159075* transcript levels failed to complement the lack of Pi uptake in *pht1;6* mutants (Figures 5, 8). It remains to be shown whether this can be explained by differences in tissue-specific expression patterns of the two genes, by contrasting affinities of the encoded proteins for Pi or different substrate specificities. In rice besides *OsPT11*, another Pi transporter gene, *OsPT13*, is enhanced upon mycorrhization and was shown to be important for arbuscular mycorrhizal colonization of the roots (Paszkowski et al., 2002; Yang et al., 2012). Based on its phylogenetic relationship and enhanced expression in MU, we hypothesize that the Pht1 protein Zm_2G159075 is the functional ortholog of OsPT13 from rice.

## Conclusions

Our results show that the maize Pi transporter Pht1;6 greatly contributes to total uptake of P in mycorrhizal maize under low P conditions in the field and under controlled conditions in the greenhouse. This transporter is therefore involved in control of biomass accumulation and cob yield. A loss-of-function of Pht1;6 coincided with impaired mycorrhizal colonization which was reconstituted in the presence of neighboring mycorrhizal plants. Fitter (2006) proposed a link between C and P fluxes in AM. In this model, the plant can locally detect increased P supply by the AMF and initiate delivery of carbohydrate to the periarbuscular space from which it is absorbed by the fungus. Since this hypothesizes that carbohydrates will only be exchanged for Pi, the fungus might not colonize *pht1;6* roots in absence of C delivery. Our observation that *trans*-complementation with mycorrhizal nurse plants restored root colonization in *pht1;6*, supports Fitter's hypothesis and alludes to the role of Pht1;6 as a molecular regulator in C for P interactions. The presence of normal arbuscules in MU doesn't stay in disagreement with Fitter's model, because it can't be excluded that residual amounts of C provided by MU to the fungus occurred via e.g., cell wall degradation through xyloglucanolytic enzymes (García-Garrido et al., 1999).

RNA sequencing suggested that a *Pht1;6* knock-out is correlated with a deregulation in hormone (GA, ABA, ethylene), signaling (receptor kinases, fatty acids) and cell structure (cell wall, lipids) pathways in mycorrhizal roots (Figure 7 and Data S1). Elucidation of the fundamental molecular mechanisms underlying these changes could reveal novel regulators of the MPU pathway and/or carbon movement to the AMF.

Given the strongly reduced cob development, and hence grain yield, in *pht1;6* on low P agricultural soil (Figure 2), it is tempting to speculate that the responsiveness of crops to AM fungi and the MPU pathway, which is most likely under the control of Pht1;6, substantially contributed to human population growth before the transformation of agriculture through the green revolution which was accompanied by input of large amounts of chemical fertilizer. Today, to satisfy the ever increasing demand for food, raw materials and bio-energy, the improvement of mycorrhiza responsiveness and MPU efficiency through e.g., increased expression of Pht1;6-like proteins exhibiting higher affinity for Pi in newly bred crop lines could help to guarantee high yield in low P input agricultural soils and thus reduce wasteful use of fertilizer.

Besides improved P uptake, AM symbiosis enhances plant resistance to pathogen infection and abiotic stress including mild drought (Whipps, 2004; Porras-Soriano et al., 2009). Therefore, selective breeding for enhanced AM responsiveness could lead to increased crop yield under a wide range of environmental conditions and, ultimately, to increased sustainability with agro-ecosystems due to a better exploitation of symbiotic relationships with soil micro-organisms. Natural variation in growth and biomass yield and AM responsiveness in maize under Pi-limiting conditions were already described and could be exploited in applied plant breeding programs including QTL mapping and marker-assisted selection (Kaeppler et al., 2000). In this context, Pht1;6 loss-of-function mutants which are mycorrhizal due to *trans*-complementation by neighboring plants could serve as AM non-responsive control plants.

## Materials and methods

### Plant material and genetic analysis

F1 and F2 seeds of plants carrying the *pht1;6::Mu* mutation used in this study were identified and produced by Pioneer Hi-Bred International, Inc. using the TUSC (Trait Utility System for Corn) technology (McCarty and Meeley, 2009). Plants were checked for Mu transposable element insertion into *Pht1;6* gene by PCR (Table S1) on leaf genomic DNA (Dellaporta et al., 1983) using gene specific primer 59772 (gcggacacctgccttacattgcc) and degenerated *Mu* terminal inverted repeat primer (MuTIR; agagaagccaacgccawcgcctcyatttcgtc). The site of Mu insertion was determined by sequencing of the resulting PCR products (Eurofins MWG Operon, Ebersberg, Germany). Quality control of DNA was carried out using 59772 and 59773 (cctggatctcgacgtccatcacct) primers (Figure 1A). In order to identify the wild type *Pht1;6* allele, primer pairs RT4 (acagcagcgggtagtcgttcg) and FT7 (gtcgtggcaggaccagatg) as well as primers 6a (catgggcgtcgcggacgtg) and 6b (ggcgtgatcacatggattcc) (Figure 1A) were used. Unless otherwise noted, 50 ng DNA was used in 25μl of PCR reaction assay using GoTaq flexi polymerase (Promega, Mannheim, Germany) according to the manual. 1.5 mM of MgCl_2_ was used per reaction. PCR was carried out in a Verity Thermal Cycler (Applied Biosystems, Carlsbad, CA, USA) using the appropriate program for the different primer combinations (Table S1). PCR products were separated on a 2% agarose gel and stained with ethidium bromide. Heterozygous (HE) *Pht1;6*/*pht1;6* mutants identified among F2 plants were backcrossed to the maize line B73 and HE plants were subsequently identified by PCR and used for further backcrosses to B73. HE plants from the progeny of each of the 2nd, 3rd, and 4th backcross were self-pollinated. The resulting segregating seeds were subsequently used for the phenotypical analyses in the field and in the greenhouse. Segregating seeds from one single cob were used within one experiment and zygosity of each seed-derived plant was identified by PCR analysis of leaf DNA as described above.

### RNA expression analysis

Total RNA from maize roots was extracted using the “hot phenol method” (Verwoerd et al., 1989) or the method provided in the “Plant RNA extraction kit” (Macherey-Nagel, Düren, Germany). DNase I (Fermentas St. Leon-Rot, Germany) treatment of 1 μg of purified total RNA was performed prior to cDNA synthesis using MVMult reverse transcriptase (Fermentas). PCR on cDNA was performed in a Veriti Thermal Cycler (Applied Biosystems) and PCR products were visualized by ethidium bromide in 2% agarose gels. For semi-quantitative PCR, gene primers specific for *Pht1;6* (RT4, FT7; 6a, 6b) and *Pht1;3* (ZmPT3-F1: caagtacgtacgtaacgtaacg; ZmPT3-R1: cgatcgcgcaagttcaactg) were used. *Ubiquitin* served as cDNA loading control (Trevisan et al., 2008).

### Statistical analyses

To test for significant differences among at least three groups One-Way ANOVA and for pair wise comparisons student's *t*-test was carried out using IBM SPSS Statistics, version 20 (IBM, Armonk, New York).

### Histochemical staining and microscopy

Roots were harvested together with the remaining stem and were extensively washed and carefully separated. Only roots that were connected to one plant root system have been used for further analyses to avoid intermingling with roots from neighboring plants. One part (approx. 100 mg fresh weight) was frozen in liquid N and stored at −20°C for RNA extraction, another part was stained for fungal hyphae according to a protocol based on Phillips and Hayman (1970) and Brundrett et al. (1984). Briefly, roots were digested in 10% KOH at 65°C for 60 min, rinsed with water, incubated in 1% HCl at room temperature for 30 min, then transferred (with no further rinsing) to 0.05% Trypan blue in lactic acid: glycerol: water (1: 1: 1, v/v/v) and stained at 95°C for 2 h. Finally, the roots were incubated overnight in glycerol: water (1: 1, v/v) at room temperature. The extent of root length colonized by hyphae, arbuscules and vesicles was determined on stained root samples according to the method of McGonigle et al. (1990), recording 150 root intersects per sample by light microscopy (Leica DM 1000 LED) at 400× magnification.

For Wheat Germ Agglutinin (WGA) Alexa Fluor 488 (New England Biolabs, Ipswich, MA, USA) staining, MU and HE roots (*n* = 3) were placed into phosphate-buffered saline containing 0.2 μg ml^−1^ WGA Alexa Fluor 488. Arbuscule size and percentage of septation in HE and MU roots (each *n* = 3) were analyzed in 150 root sections with a fluorescence microscope DM5000B (Leica, Leica Microsystems, Wetzlar, Germany) using a 63× HCX PL APO CS oil-objective NA1,4 and were quantified using the “LAS AF” software (Leica). A confocal fluorescence microscope (Leica TCS SPE DM5500, 63× ACS APO oil-objective NA1,3; 2 μm steps) was used to visualize fluorescent signals. Fungal cell wall (WGA Alexa Fluor 488-stained) and plant cell wall autofluorescence were detected using 510–540 nm and 565–615 nm emission filters, respectively.

### Experimental field trials

Experimental field trials were carried out at the ART in the vicinity of Zürich (Switzerland) during three years (2006, 2007, and 2009). The experimental area is 443 m above sea level, long-term average annual rainfall is 1042 mm, long-term average annual temperature is 8.5°C, and consists mostly of a deep cambisol. The upper floor has a humus content of 2.5–3% and a clay content of 20–25%. Since 1989 separated field segments were not fertilized at all (−[PNK]), or were fertilized with nitrogen (N), P and potassium (K) (+[PNK]) or with N and K without P (−[P] +[NK]) (Gallet et al., 2003; Hausherr et al., 2007). Each field segment is 8 × 5 m in size. A 7-year crop rotation plan has been applied (two different leys, wheat, sugar beet, maize, potato, and barley). The tillage with plow, harrow, hoe, and milling machine was culture-dependent and identical for all fertilizer regimes. Plant protection measures were chosen to minimize the influence of plant diseases and pests on plant fitness. Concentration of plant available and total minerals within the soil of each field is measured every year (Hons et al., 1990). Mineral fertilization was performed in general agreement with the soil test and crop requirement. At time of harvest, length of plant shoots and cob status was recorded. Plant shoots were separated into leaves, stems and cobs, then dried at 65°C to constant weight and subsequently ground. For quantification of K, Ca, Mg, P, Fe, Cu, Zn, one g of plant dry material of the respective organs was microwave-digested with nitric acid and analyzed by an Agilent 7500 Series ICP-MS.

### Mycorrhizal pot experiments

Experiments were carried out in a greenhouse at 16 h of light (Son-T MASTER Agro 400W; Philips GmbH, Hamburg, Germany) and at 24°C/21°C (light-/dark-phase). Commercially available seeding compost (Stender Vermehrungssubstrat A210, Stender, Germany) and quartz sand (0.71–1.25 mm; Quarzwerke GmbH, Frechen, Germany) had been autoclaved two times for 20 min at 121°C, air dried and thoroughly mixed in the ratio 1:9 (v/v) prior to be used for cultivation. Arbuscular mycorrhizal fungi (AMF) were added using 75 g dry-inoculum (*Plantago lanceolata* roots colonized by *Glomus intraradices* (syn: *Rhizophagus irregularis*), BEG75) per kg of dry mixed substrate (1:9), while for the non-mycorrhizal conditions, AMF were omitted. Pots (with small drainage holes in the bottom) were fertilized with single-strength Hoagland solution (Hoagland and Broyer, 1936) containing diammonium hydrogen phosphate (10 μM, “low Pi”) and ammoniumchloride (1 mM) as P and N sources. In order to maintain a water-holding-capacity of 70–80%, compartments were watered with de-ionized water to weight. Two seeds per pot were planted, genotypes of the germinated plants were determined by PCR and one of each seedling pair was removed in order to end up with four replicates of each genotype.

For the usage of chive nurse plants, *Allium schoenoprasum* was pre-grown in a greenhouse for 8 weeks in mother pots containing quartz/ soil mixture (9:1 v/v) with or without added *Glomus intraradices* and fertilized with half-strength “low Pi” Hoagland solution. Single mycorrhizal and non-mycorrhizal chive plants were then transferred to new pots each containing 1800 g of quartz/soil mixture. After 5 weeks of chive cultivation, the maize seeds (derived from 4th backcross) were sown and fertilization with single strength “low Pi” Hoagland solution (3 × 50 ml per week) started 1 week post-planting.

### Radiolabeled phosphate uptake study

First, chive nurse plants were pre-grown and pots were prepared as described above. A scintillation vial was filled with the above described soil mixture, capped with a 25 μm nylon mesh (Nagy et al., 2005) and served as hyphal compartment (HC). A 2 mm pinhole was punched into the bottom of the vial. In contrast to the method described in Nagy et al. (2005), the vessel was buried in the soil substrate with the capped lid downwards in a way that the pinhole in the bottom of the vessel was accessible (about 0.5 cm above the soil surface). Pots were fertilized with single-strength Hoagland solution (Hoagland and Broyer, 1936) as described above and were watered to weight to 70–80% of water holding capacity using de-ionized water. After 5 weeks of chive cultivation, the maize seeds (derived from 4th backcross) were sown and the pots were placed in a growth chamber (Percival PGC-6L; Plant Climatics, Wertlingen, Germany) at 16 h light at 24°C/21°C (light-/dark-phase) and at a relative humidity of 70%. Maize genotypes were detected by PCR as described above. After 6 weeks of co-culture root samples were extracted from pots using a cork borer and AMF colonization was subsequently assessed as described above. Subsequently, 270 kBq of carrier-free ^33^P labeled H_3_PO_4_ (Hartmann Analytik GmbH, Braunschweig, Germany) was injected into each hyphal compartment (8.2 kBq/g) through the pinhole in the bottom. Two weeks post-injection, plant material (maize and chive leaves of *n* = 3 plants of each treatment) was harvested and dried at 65°C to constant weight. Subsequently, dry plant material was digested at 100°C in a 6:2 (v/v) solution of HNO_3_/H_2_O_2_. An aliquot of this digest was mixed with scintillation cocktail (Rotiszint eco plus, Roth, Karlsruhe, Germany) and was used for detection of ^33^P signals using a scintillation counter (Beckman Coulter LS 6500).

### Radiolabeled phosphate leaf translocation studies

Translocation of ^33^P in maize shoots was studied following Rausch et al. (2004). AZ, HE, and MU plants were cultivated in pure quartz sand in a phytochamber (Johnson Controls, 16 h/8 h light/dark phase at 24°C/21°C, and relative humidity of 60%) and were fertilized with single strength Hoagland solution without Pi or with Pi (1 mM) respectively. Three to 4 weeks old plants were treated with carrier-free ^33^P labeled H_3_PO_4_ (Hartmann Analytik GmbH, Braunschweig, Germany), i.e., a set of plants was used for Pi translocation studies at the shoot level, and another set was used to study Pi transport from root to shoot. For translocation studies within the shoot a pipette tip was used to pick two holes into a fully developed leaf and twice 2 μl of labeled ^33^P were applied (200 kBq total activity). When ^33^P droplets were completely absorbed by the leaf, twice 2 μl water were added. After 24 h leaves were harvested, dried and measured in a scintillation counter as described before. For uptake studies via the root quartz/soil substrate was removed carefully and plants were transferred into 100 ml of corresponding Hoagland solution. After 1 h of adaption 200 kBq were added to the solution. Harvesting of leaves was done 24h after ^33^P-application. After drying of the leaf material analysis of ^33^P-tracer accumulation was performed in a scintillation counter as described before.

### RNA sequencing and mapping of transcriptome

Experiments were carried out in a greenhouse as described above. Mycorrhizal and non-mycorrhizal chive nurse plants were pre-grown and the HE and MU maize seeds (derived from 4th backcross) were sown and pots were fertilized using low Pi Hoagland solution as described above. Maize roots were harvested after 8 weeks of co-culture (*n* = 3 per treatment). Total RNA was extracted using standard RNeasy Plant Mini Kit (Qiagen GmbH, Hilden, Germany). RNA purity was checked via photometric measurements using NanoDrop 1000 (Thermo Fisher Scientific Inc., Waltham, MA, USA) and RNA integrity was analyzed by gel electrophoresis using Agilent 2100 Bioanalyzer (Agilent Technologies GmbH, Boeblingen, Germany RNA 6000 Nano Kit).

Upon quality assessments of the 12 total RNA samples, ≥1 μg total RNA was used for TruSeq™ RNA sample preparation (Illumina Inc., San Diego, CA, USA). PolyA-enriched, enzymatically fragmented total RNA of a size of 200–300 bp was reverse transcribed and ligated to barcoded adapters to construct sequencing libraries. Libraries were validated and six libraries were pooled for sequencing on one channel. Sequencing was performed on an Illumina Genome Analyzer IIx with 100 bp paired-end (PE) reads resulting in an expected data volume of 30 gigabyte per lane which corresponds to approx. One hundred and fifty million paired-end reads. Each sample was represented by 20–30 million PE reads in average corresponding to 700 hits/gene at a genome size of 35.000 genes.

High quality short-reads were mapped to the *Zea mays* genome sequence (Release 5b.60, assembly version AGPv2) from “www.maizesequence.org” using default parameters on Burrows-Wheeler Aligner (BWA) (Li and Durbin, 2010). The hits per gene were counted using HTSeq-count from Simon Anders, EMBL Heidelberg (www-huber.embl.de/users/anders/HTSeq/), splice variants of one gene were summed up and differential expression of genes was analyzed by negative binomial statistics using DESeq (Anders and Huber, 2010). Genes with fold change ≥2.0 with a *p* ≤ 0.05 (adjusted for multiple testing by the method of Benjamini and Hochberg (1995), were chosen as differentially expressed genes. Comparison of maize genes of the working_set_5b (http://maizesequence.org) to *Arabidopsis thaliana* genome database version TAIR10_pep_20101214 (www.arabidopsis.org) was performed by protein blast analysis (Altschul et al., 1997) and best hit (below a threshold of 1e-10) was taken for annotations in Data S1. Gene set enrichment analyses were performed using Fishers Exact Test (adjusted for multiple testing by the method of Benjamini and Hochberg (1995) using “MAPMAN Bin codes” (Thimm et al., 2004)from mapping file “Zm_B73_5b_FGS_cds_2011” (http://mapman.gabipd.org).

## Author contributions

Martin Willmann conceived and carried out the experiment, designed and carried out the data analysis, and co-wrote the paper. Nina Gerlach carried out the experiment and carried out the data analysis, and co-wrote the paper. Benjamin Buer carried out RNA-Seq experiment and data analysis and co-wrote the paper. Aleksandra Polatajko carried out the ICP-MS and data analysis. Réka Nagy conceived and carried out the initial steps in the experiment. Eva Koebke carried out the experiment regarding arbuscular size classes and septation. Jan Jansa carried out the experiment regarding mycorrhizal root colonization in the field. René Flisch conceived the field experiment and co-wrote the paper. Marcel Bucher conceived the experiment, designed the data analysis and co-wrote the paper.

### Conflict of interest statement

The authors declare that the research was conducted in the absence of any commercial or financial relationships that could be construed as a potential conflict of interest.

## Supplementary material

Figure S1 **Detection of *Pht1;6* transcripts via RT-PCR.** RNA was isolated from two individual plants each of MU, HE, and AZ mature leaves and mycorrhizal roots of segregants of 2nd backcross taken from the -P [+NK] field. *Pht1;6* primers FT7/RT4 are transposon flanking primers, while 6a/6b are 3' downstream of the transposon insertion site of the *Pht1;6* gene. *Ubiquitin* transcripts were used as cDNA normalization control.

Figure S2 **Growth of shoot and cobs of *pht1;6* mutant maize in the field.** For field trials maize seeds were produced by several back-crosses of *pht1;6* plants to B73. After each round of backcross self-pollination was carried out, in order to receive a seed population exhibiting segregation of *pht1;6::Mu*. Segregating seeds from a single cob of the 2nd and 3rd backcross were sown in spring (2006 and 2007, respectively) and were grown during a vegetation period of about 6 months under field conditions as described in “Material and Methods.” The status of zygosity with respect to *pht1;6::Mu* (MU = homozygous; HE = heterozygous; AZ = azygous) was detected using PCR on genomic DNA of leaves (*n* see Table S2). Means and standard deviations of shoot **(A)** and cob **(B)** dry weight of plants with segregating *pht1;6::Mu* and grown under the indicated field conditions are shown. Experiments not performed in 2006 are indicated (n.p.). n.d., indicates “not detected.” Significant differences of mean values between groups of treatment within one genotype (*n* see Table S2) for *p* < 0.05 were determined by One-Way ANOVA analysis and are indicated by asterisks.

Figure S3 **Changes in elemental profiles in shoots of field grown maize.** Segregating maize seeds originating from a single cob of the 2nd and 3rd and backcross were sown in spring 2006 and 2007 and were grown on fields as described in “Materials and Methods” and in Figure S2. For description of genotypes see legend to Figure S2. Mean values of element concentrations of MU shoots were divided by the mean values of HE or AZ shoots to indicate fold change of MU vs. HE/AZ. Significant differences of mean values (*p* < 0.05, for *n* see Table S2) used for calculating the ratio were determined by One-Way ANOVA analysis and are indicated by large asterisks when MU/HE and MU/AZ ratios both are significantly different or small when only MU/AZ ratios are significantly different. Dashed line at *y* = 1 means “no fold change.”

Figure S4 **P concentration in tissues of plants grown on +[NPK] fields.** Segregating maize seeds originating from a single cob of the 3rd and 4th backcross were sown in spring 2007 and 2009 and were grown on +[NPK] fields as described in “Materials and Methods” and in Figure S2. For description of genotypes see legend to Figure S2. Means and standard deviations of P concentrations in separate maize tissues (dry weight, DW) are shown. Significant differences of mean values within groups of one treatment (*n* see Table S2) for *p* < 0.05 were determined by One-Way ANOVA analysis and are indicated by asterisks (^*^).

Figure S5 **Translocation of ^33^P within the maize shoot.** ^33^P was applied on the first fully developed leaf of heterozygous (HE) and mutant (MU) plants grown at Pi-deficient (−Pi) and Pi-sufficient (+Pi) conditions. Distribution of radioactivity within the shoot was followed up after 24 h **(A)** via scintillation counting (*n* = 3, Students *t*-test, *p* ≤ 0.05) and **(B)** via phosphorimager analysis. **(C)** Schematic depiction of a maize plant which illustrates leaf numbering and application of ^33^P.

Figure S6 **P concentration in shoots and roots of maize plants used for transcriptomics studies.** MU and HE plants (each *n* = 3) derived from 4th backcross were grown in single pots together with mycorrhizal (*G. intraradices*, BEG75) chive nurse plants (+myc) or chive without AMF (−myc) for 8 weeks at low Pi conditions. Means and standard deviations of P concentrations detected in shoots and roots of MU and HE maize plants are shown. Significant differences of mean values between groups of treatment within one genotype were determined by One-Way ANOVA analysis (*p* < 0.05) and are indicated by either asterisks or letters. Means and bars with the same letter are not significantly different. n.d., not detected.

Figure S7 **Nucleotide fragments mapped to *Pht1;6* in transcriptomic analyses.** Short reads of *Pht1;6* of MU and HE samples mapped to the *Pht1;6* sequence using BlastN with similar parameters like BWA (2 mismatches, 0 gap openings). Hits of *Pht1;6* from MU plant samples map to frequently mapped regions in HE plants suggesting conserved motifs in all members of the *Pht1* gene family.

Table S1 **Segregation of *pht1;6::Mu* in field trials.** Segregation of *pht1;6::Mu* in plants from field trials after backcross (BC) to B73 and self pollination (S). Plants used for the field trials originated from seeds of one single cob for each generation of backcross/year. Chi Square statistics confirmed Mendelian segregation of the *pht1;6::Mu* mutation.

Table S2 **PCR conditions with each primer pair used for genetic analysis**.

Table S3 **Concentrations of extractable P and K in the lots used for field trials at ART.** Method used at ART Research Station was soil extraction with ammonium acetate EDTA (AAE10) (Hons et al., 1990).

Table S4 **Elemental profiles in shoots of MU, HE, and AZ maize plants.** Segregating maize seeds originating from a single cob of the 4th backcross were sown in spring 2009 and grown on fields as described in “Materials and Methods” and in Figure S2. For description of genotypes see legend to Figure S2. Averages and standard deviations of element amounts per shoot dry weight [mg/kg] of plants grown under the indicated field conditions are shown. Significant differences of mean values between groups of treatment within one genotype (*n* see Table S2) for *p* < 0.05 were determined by One-Way ANOVA analysis and are indicated by asterisks (^*^).
