# Supplementary material for: Mycorrhizal phosphate uptake pathway in maize: vital for growth and cob development on nutrient poor agricultural and greenhouse soils
Source: Front Plant Sci. 2013 Dec 26;4:533. doi: 10.3389/fpls.2013.00533 (PMC3872827; doi:10.3389/fpls.2013.00533)
Supplement: Figure S1 — Detection of Pht1;6 transcripts via RT-PCR. RNA was isolated from two individual plants each of MU, HE, and AZ mature leaves and mycorrhizal roots of segregants of 2nd backcross taken from the -P [+NK] field. Pht1;6 primers FT7/RT4 are transposon flanking primers, while 6a/6b are 3' downstream of the transposon insertion site of the Pht1;6 gene. Ubiquitin transcripts were used as cDNA normalization control. [file DataSheet2.PDF]

LEGENDS FOR SUPPORTING INFORMATION

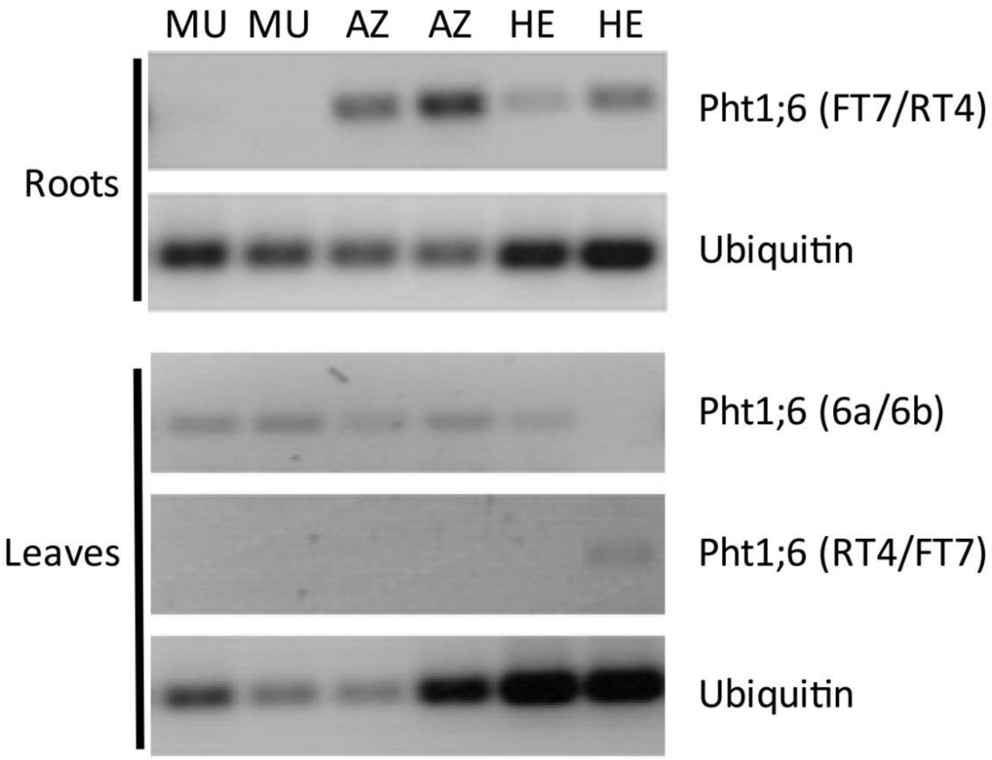

Figure S1. Detection of *Pht1;6* transcripts via RT-PCR. RNA was isolated from two individual plants each of MU, HE and AZ mature leaves and mycorrhizal roots of segregants of 2nd backcross taken from the -P [+NK] field. *Pht1;6* primers FT7/RT4 are transposon flanking primers, while 6a/6b are 3' downstream of the transposon insertion site of the *Pht1;6* gene. *Ubiquitin* transcripts were used as cDNA normalization control.

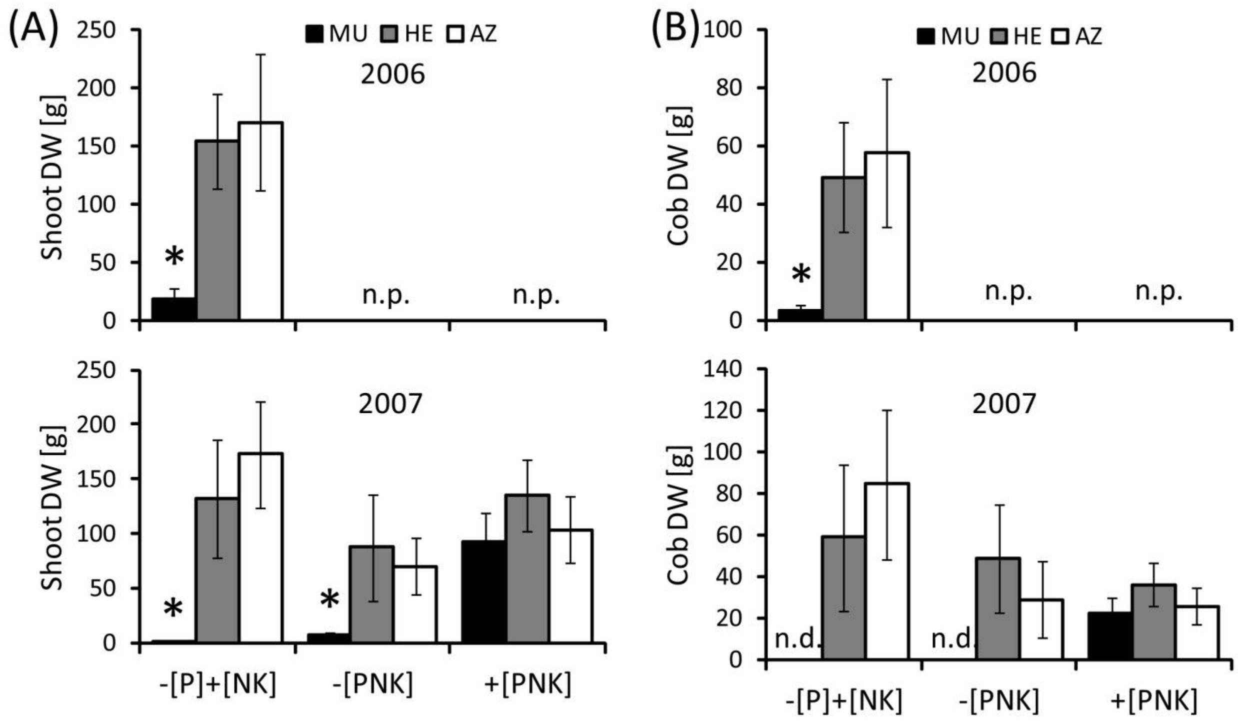

Figure S2. Growth of shoot and cobs of *pht1;6* mutant maize in the field. For field trials maize seeds were produced by several back-crosses of *pht1;6* plants to B73. After each round of backcross self-pollination was carried out, in order to receive a seed population exhibiting segregation of *pht1;6::Mu*. Segregating seeds from a single cob of the 2nd and 3rd backcross were sown in spring (2006 and 2007, respectively) and were grown during a vegetation period of about 6 months under field conditions as described in "Material and Methods". The status of zygosity with respect to *pht1;6::Mu* (MU=homozygous; HE=heterozygous; AZ=azygous) was detected using PCR on genomic DNA of leaves (*n* see Table S2). Means and standard deviations of shoot (A) and cob (B) dry weight of plants with segregating *pht1;6::Mu* and grown under the indicated field conditions are shown. Experiments not performed in 2006 are indicated (n.p.). n.d. indicates "not detected". Significant differences of mean values between groups of treatment within one genotype (*n* see Table S2) for $p < 0.05$ were determined by one-way ANOVA analysis and are indicated by asterisks.

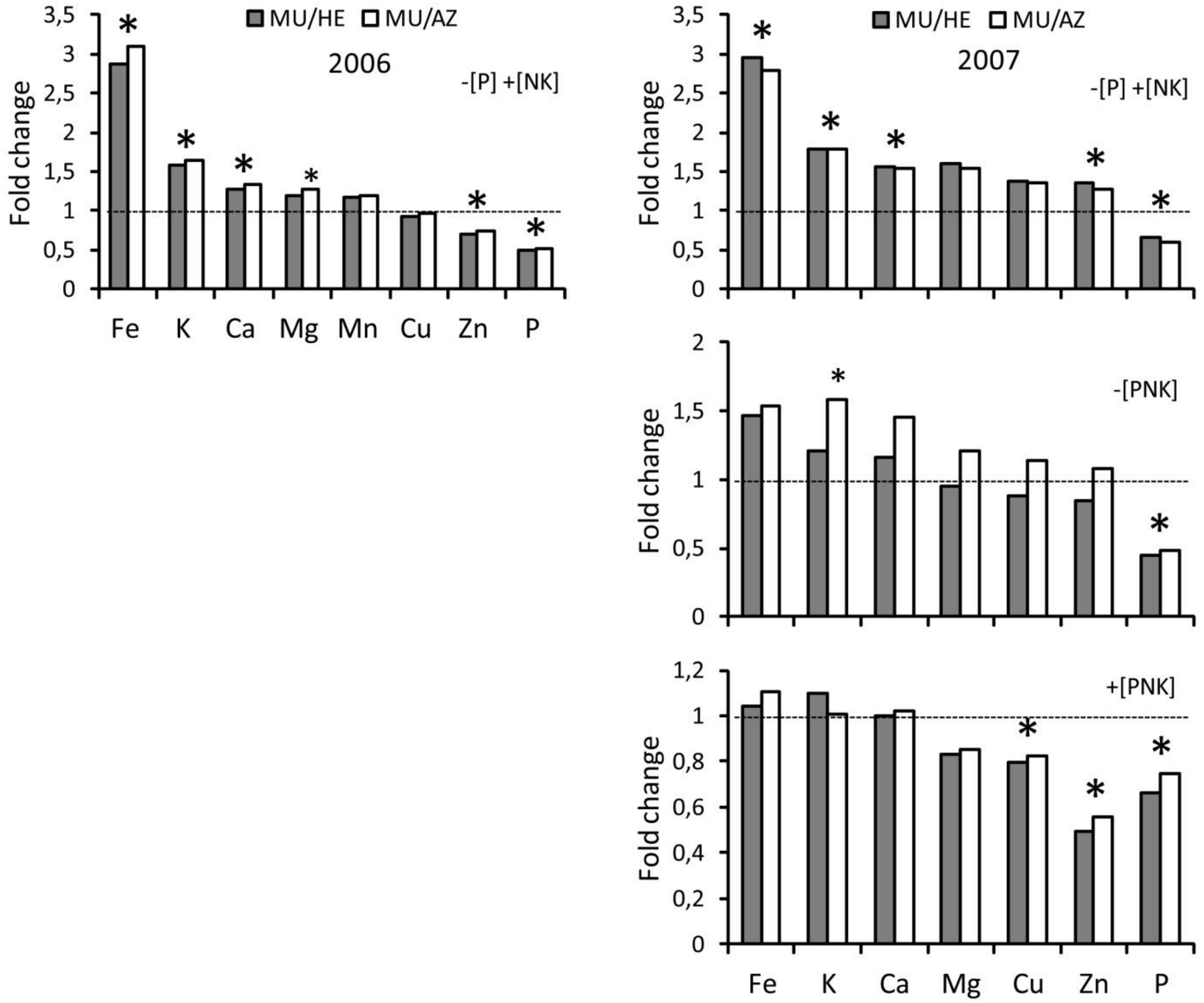

Figure S3. Changes in elemental profiles in shoots of field grown maize. Segregating maize seeds originating from a single cob of the 2nd and 3rd and backcross were sown in spring 2006 and 2007 and were grown on fields as described in "Materials and Methods" and in Figure S2. For description of genotypes see legend to Figure S2. Mean values of element concentrations of MU shoots were divided by the mean values of HE or AZ shoots to indicate fold change of MU vs. HE/AZ. Significant differences of mean values (p < 0.05,for n see Table S2) used for calculating the ratio were determined by one-way ANOVA analysis and are indicated by large asterisks when MU/HE and MU/AZ ratios both are significantly different or small when only MU/AZ ratios are significantly different. Dashed line at y=1 means "no fold change".

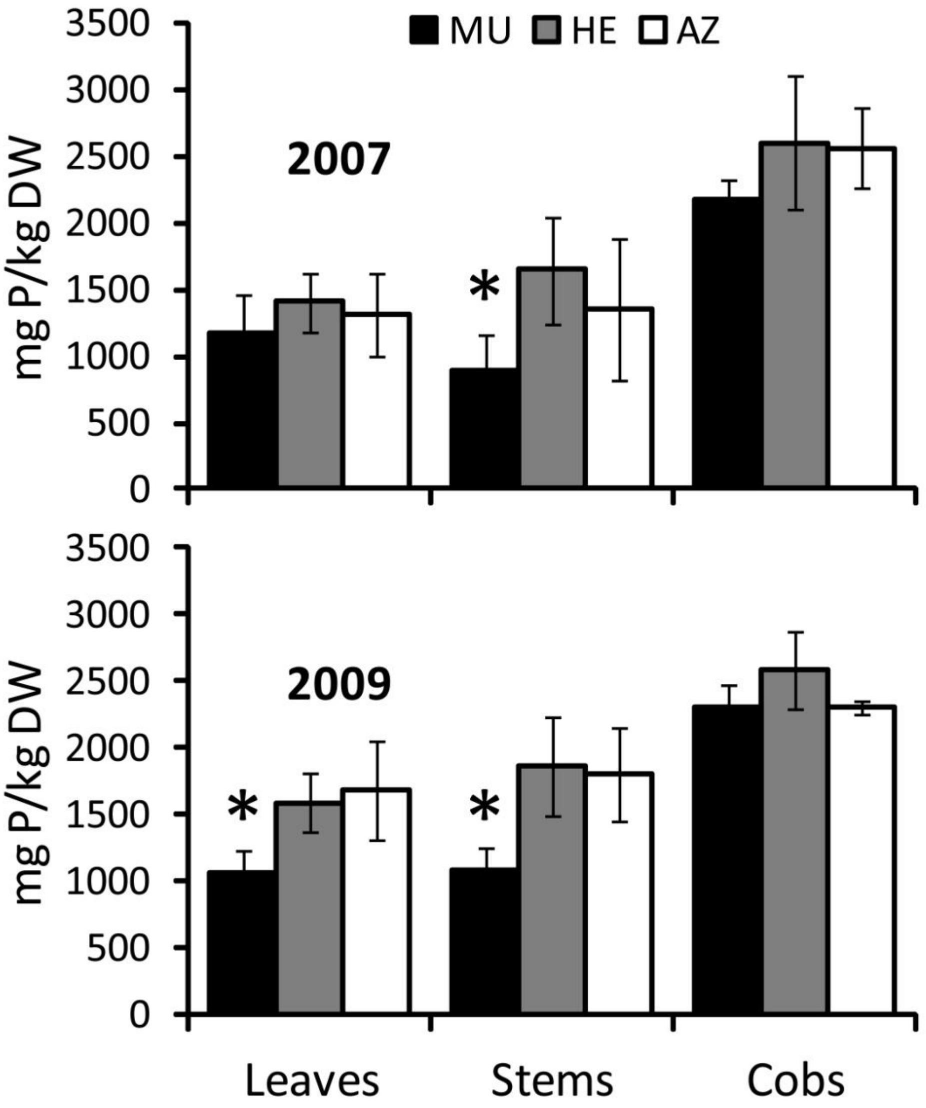

Figure S4. P concentration in tissues of plants grown on +[NPK] fields. Segregating maize seeds originating from a single cob of the 3rd and 4th backcross were sown in spring 2007 and 2009 and were grown on +[NPK] fields as described in "Materials and Methods" and in Figure S2. For description of genotypes see legend to Figure S2. Means and standard deviations of P concentrations in separate maize tissues (dry weight, DW) are shown. Significant differences of mean values within groups of one treatment (*n* see Table S2) for $p < 0.05$ were determined by one-way ANOVA analysis and are indicated by asterisks (*).

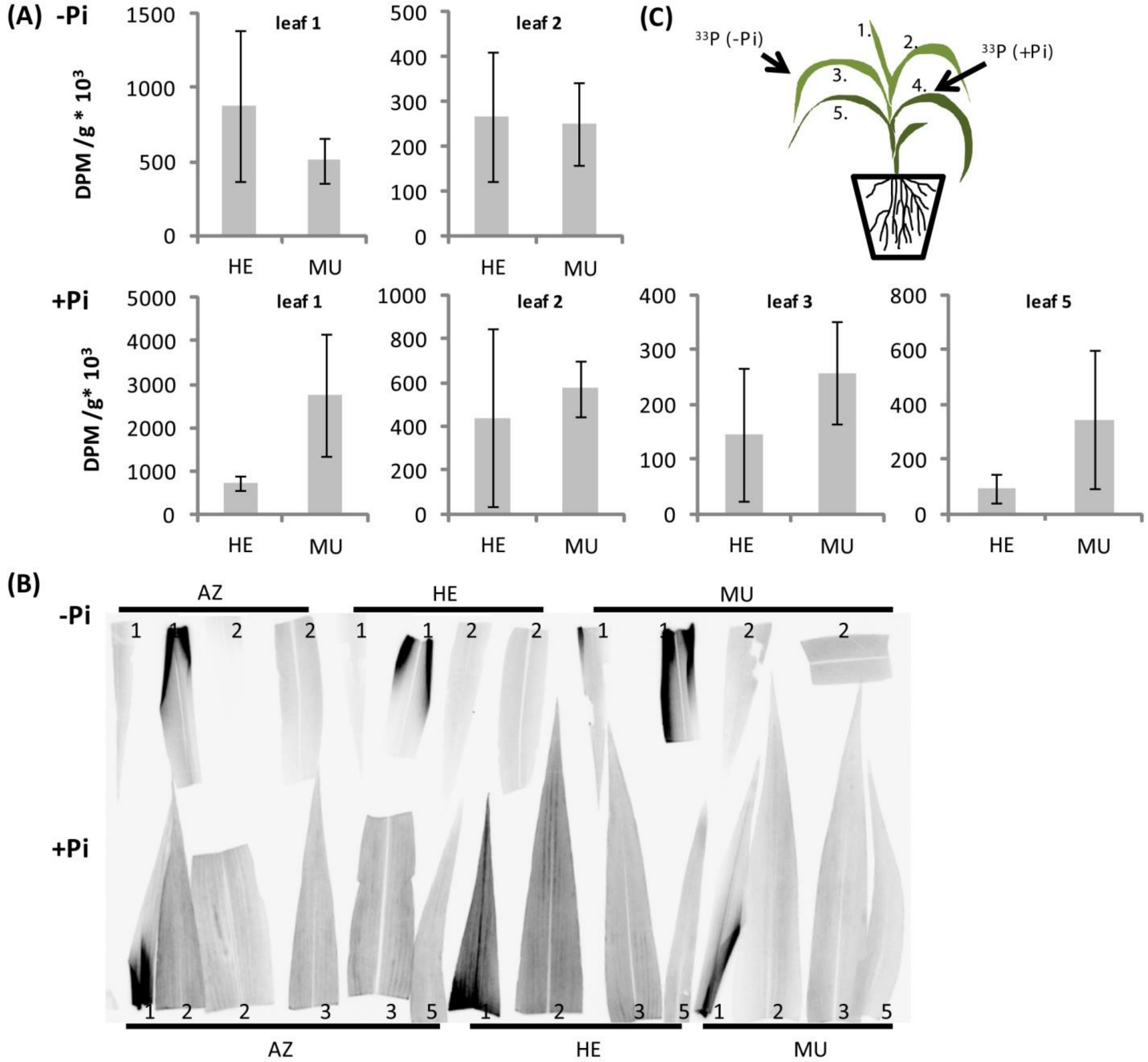

Figure S5: Translocation of ^{33}P within the maize shoot. ^{33}P was applied on the first fully developed leaf of heterozygous (HE) and mutant (MU) plants grown at Pi-deficient (-Pi) and Pi-sufficient (+Pi) conditions. Distribution of radioactivity within the shoot was followed up after 24 hours (A) via scintillation counting (n = 3, Students t-test, $p \leq 0.05$) and (B) via phosphorimager analysis. (C) Schematic depiction of a maize plant which illustrates leaf numbering and application of ^{33}P.

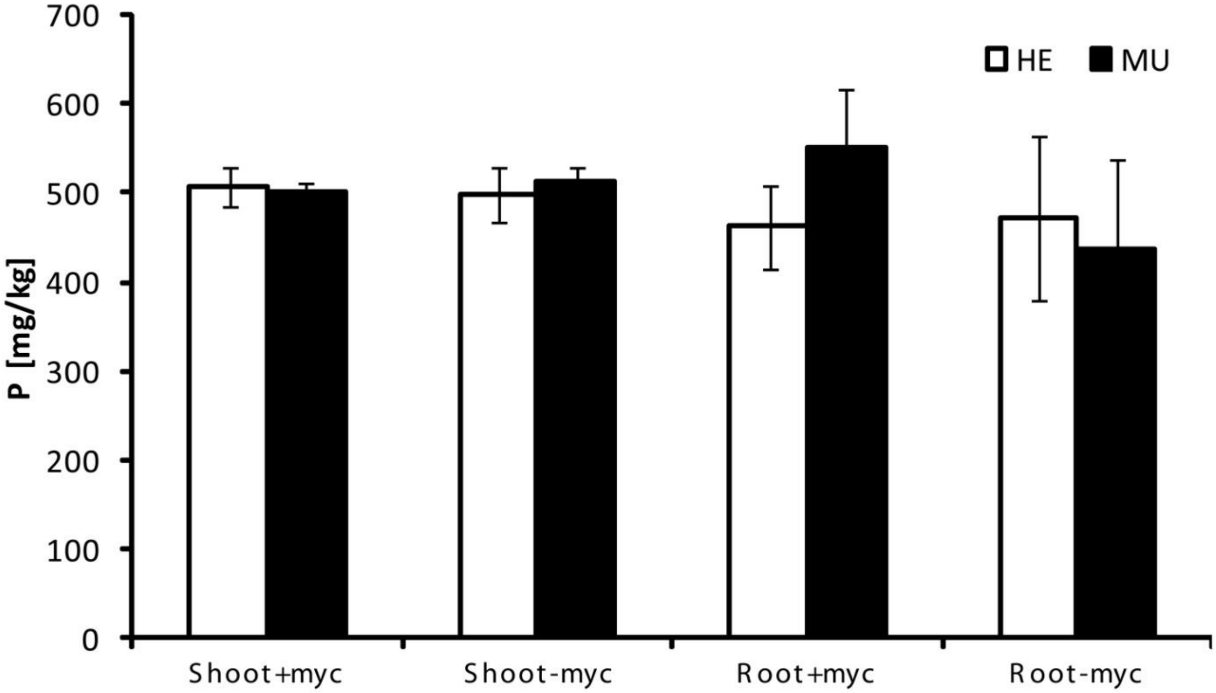

Figure S6. P concentration in shoots and roots of maize plants used for transcriptomics studies. MU and HE plants (each n=3) derived from 4th backcross were grown in single pots together with mycorrhizal (*G. intraradices*, BEG75) chive nurse plants (+myc) or chive without AMF (-myc) for 8 weeks at low Pi conditions. Means and standard deviations of P concentrations detected in shoots and roots of MU and HE maize plants are shown. Significant differences of mean values between groups of treatment within one genotype were determined by one-way ANOVA analysis ($p < 0.05$) and are indicated by either asterisks or letters. Means and bars with the same letter are not significantly different. n.d.= not detected.

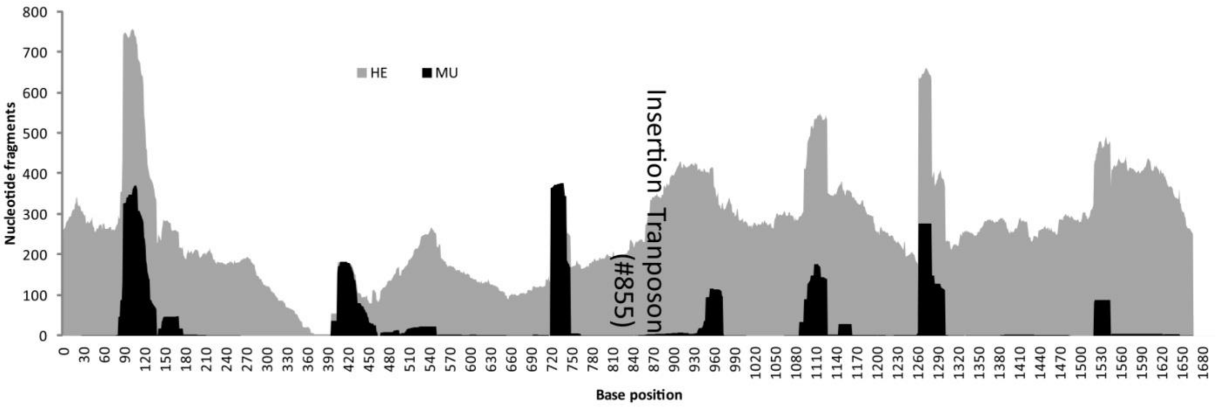

Figure S7. Nucleotide fragments mapped to *Pht1;6* in transcriptomic analyses. Short reads of *Pht1;6* of MU and HE samples mapped to the *Pht1;6* sequence using BlastN with similar parameters like BWA (2 mismatches, 0 gap openings). Hits of *Pht1;6* from MU plant samples map to frequently mapped regions in HE plants suggesting conserved motifs in all members of the *Pht1* gene family.
