# Supplementary material for: Mycorrhizal phosphate uptake pathway in maize: vital for growth and cob development on nutrient poor agricultural and greenhouse soils
Source: Front Plant Sci. 2013 Dec 26;4:533. doi: 10.3389/fpls.2013.00533 (PMC3872827; doi:10.3389/fpls.2013.00533)
Supplement: Table S1 — Segregation of pht1;6::Mu in field trials. Segregation of pht1;6::Mu in plants from field trials after backcross (BC) to B73 and self pollination (S). Plants used for the field trials originated from seeds of one single cob for each generation of backcross/year. Chi Square statistics confirmed Mendelian segregation of the pht1;6::Mu mutation. [file DataSheet3.PDF]

Table S1. Segregation of *pht1;6::Mu* in field trials. Segregation of *pht1;6::Mu* in plants from field trials after backcross (BC) to B73 and self pollination (S). Plants used for the field trials originated from seeds of one single cob for each generation of backcross/year. Chi Square statistics confirmed Mendelian segregation of the *pht1;6::Mu* mutation.

Seeds	Field condition	*Pht1;6/Pht1;6* (AZ)	*Pht1;6/pht1;6* (HE)	*pht1;6/pht1;6* (MU)	Ratio AZ/HE/MU
BC2 x S	-[P] +[NK]	8	14	8	1 / 1.8 / 1
BC3 x S	-[P] +[NK]	12	23	3	1 / 1.9 / 0.25
BC3 x S	-[PNK]	10	21	4	1 / 2.1 / 0.4
BC3 x S	+[PNK]	10	25	7	1 / 2.5 / 0.7
BC3 x S	Total	32	69	14	1 / 2.2 / 0.4
BC4 x S	-[P] +[NK]	8	12	6	1 / 1.5 / 0.75
BC4 x S	-[PNK]	10	11	4	1 / 1.1/ 0.4
BC4 x S	+[PNK]	4	16	7	1 / 4 /1.75
BC4 x S	Total	22	39	17	1 / 1.8 / 0.8
BC2,3,4 x S	All	62	122	39	1 / 2 / 0.6

Table S2. PCR conditions with each primer pair used for genetic analysis.

59772/59773; 59772/MuTIR		***6a/6b; RT4/FT7***		
3 min.	94°C	3 min.	94°C	
1 min.	94 °C	1 min.	94 °C	34 cycles
45 sec.	58 °C	30 sec.	63 °C	
1 min.	72 °C	1 min.	72 °C	
10 min.	72°C	10 min.	72°C	

Table S3. Concentrations of extractable P and K in the lots used for field trials at ART. Method used at ART Research Station was soil extraction with ammonium acetate EDTA (AAE10) (Hons et al., 1990).

Date	Conditions	P (mg/kg)	K (mg/kg)
**11/2005**	-[P] +[NK]	6.8	141.6
**01/2007**	-[P] +[NK]	6.4	143.8
**12/2007**	-[P] +[NK]	6.9	147.7
**12/2008**	-[P] +[NK]	7.7	131.3
**12/2009**	-[P] +[NK]	6.7	126.1
**11/2005**	-[PNK]	9.2	47.5
**01/2007**	-[PNK]	9.4	58.5
**12/2007**	-[PNK]	9.9	62.1
**12/2008**	-[PNK]	10	51.7
**12/2009**	-[PNK]	9.6	52.0
**11/2005**	+[PNK]	38	132.4
**01/2007**	+[PNK]	39.9	139.8
**12/2007**	+[PNK]	39.2	164
**12/2008**	+[PNK]	39.2	139.3
**12/2009**	+[PNK]	35.7	131.9

Table S4. Elemental profiles in shoots of MU, HE and AZ maize plants. Segregating maize seeds originating from a single cob of the 4th backcross were sown in spring 2009 and grown on fields as described in "Materials and Methods" and in Figure S2. For description of genotypes see legend to Figure S2. Averages and standard deviations of element amounts per shoot dry weight [mg/kg] of plants grown under the indicated field conditions are shown. Significant differences of mean values between groups of treatment within one genotype (*n* see Table S2) for $p < 0.05$ were determined by one-way ANOVA analysis and are indicated by asterisks (*).

		**Fe** [mg/kg]		**K** [mg/kg]		**Ca** [mg/kg]		**Mg** [mg/kg]		**Cu** [mg/kg]		**Zn** [mg/kg]		**P** [mg/kg]	
		Average	Stdv.	Average	Stdv.	Average	Stdv.	Average	Stdv.	Average	Stdv.	Average	Stdv.	Average	Stdv.
-[P]+[NK]	MU	147,63 ^{a}	23,39	22553,14 ^{a}	2273,89	3226,98 ^{a}	401,12	1274,06 ^{a}	186,99	7,50	2,52	37,72 ^{a}	14,21	613,09 ^{a}	152,21
-[PNK]	MU	173,41 ^{a}	46,97	8719,77 ^{b}	3605,98	5511,52 ^{b}	1027,97	2144,53 ^{b}	370,28	5,92	1,73	29,67 ^{a}	6,57	1042,72 ^{b}	250,36
+[PNK]	MU	45,77 ^{b}	7,84	11934,09 ^{b}	928,98	3129,03 ^{a}	194,95	1638,99 ^{c}	67,33	4,77	0,30	12,34 ^{b}	2,67	1496,09 ^{c}	160,70
-[P]+[NK]	HE	47,95 ^{a}	18,13	11458,51 ^{a}	1989,45	2679,42 ^{a}	348,75	1167,31 ^{a}	155,06	5,83	0,89	30,48 ^{a}	5,11	1587,04 ^{a}	241,61
-[PNK]	HE	68,90 ^{b}	20,56	5139,23 ^{b}	1756,59	4482,96 ^{b}	1544,20	2570,18 ^{b}	686,96	6,05	0,91	32,10 ^{a}	7,16	1974,42 ^{b}	306,00
+[PNK]	HE	40,79 ^{a}	13,14	11494,69 ^{a}	1834,02	3200,96 ^{a}	356,24	1689,47 ^{c}	131,91	5,63	0,41	17,83 ^{b}	3,62	2122,83 ^{b}	213,66
-[P]+[NK]	AZ	45,09	8,83	11895,91 ^{a}	1424,14	2439,33 ^{a}	242,00	1115,42 ^{a}	147,23	6,01	1,20	32,65 ^{a}	6,32	1788,54	361,75
-[PNK]	AZ	60,75	15,22	4611,97 ^{b}	1174,62	4087,34 ^{b}	755,84	2355,74 ^{b}	413,23	5,96	0,63	33,76 ^{a}	3,08	1949,78	133,94
+[PNK]	AZ	44,06	15,10	11170,50 ^{a}	1768,62	2938,10 ^{a}	317,98	1612,87 ^{c}	100,63	5,86	0,88	19,32 ^{b}	1,70	1992,22	153,34
